# Development and Validation of a Novel 11-Gene Prognostic Model for Serous Ovarian Carcinomas Based on Lipid Metabolism Expression Profile

**DOI:** 10.3390/ijms21239169

**Published:** 2020-12-01

**Authors:** Mingjun Zheng, Heather Mullikin, Anna Hester, Bastian Czogalla, Helene Heidegger, Theresa Vilsmaier, Aurelia Vattai, Anca Chelariu-Raicu, Udo Jeschke, Fabian Trillsch, Sven Mahner, Till Kaltofen

**Affiliations:** 1 Department of Obstetrics and Gynecology, University Hospital, LMU Munich, Maistrasse 11, 80337 Munich, Germany; Mingjun.Zheng@med.uni-muenchen.de (M.Z.); heather.mullikin@gmail.com (H.M.); Anna.Hester@med.uni-muenchen.de (A.H.); Bastian.Czogalla@med.uni-muenchen.de (B.C.); Helene.Heidegger@med.uni-muenchen.de (H.H.); Theresa.Vilsmaier@med.uni-muenchen.de (T.V.); Aurelia.Vattai@med.uni-muenchen.de (A.V.); Anca.Chelariuraicu@med.uni-muenchen.de (A.C.-R.); Udo.Jeschke@med.uni-muenchen.de (U.J.); Fabian.Trillsch@med.uni-muenchen.de (F.T.); Sven.Mahner@med.uni-muenchen.de (S.M.); 2 Department of Obstetrics and Gynecology, University Hospital Augsburg, Stenglinstrasse 2, 86156 Augsburg, Germany

**Keywords:** ovarian neoplasms, lipid metabolism, genes, The Cancer Genome Atlas (TCGA), Gene Expression Omnibus (GEO)

## Abstract

(1) Background: Biomarkers might play a significant role in predicting the clinical outcomes of patients with ovarian cancer. By analyzing lipid metabolism genes, future perspectives may be uncovered; (2) Methods: RNA-seq data for serous ovarian cancer were downloaded from The Cancer Genome Atlas and Gene Expression Omnibus databases. The non-negative matrix factorization package in programming language R was used to classify molecular subtypes of lipid metabolism genes and the limma package in R was performed for functional enrichment analysis. Through lasso regression, we constructed a multi-gene prognosis model; (3) Results: Two molecular subtypes were obtained and an 11-gene signature was constructed (PI3, RGS, ADORA3, CH25H, CCDC80, PTGER3, MATK, KLRB1, CCL19, CXCL9 and CXCL10). Our prognostic model shows a good independent prognostic ability in ovarian cancer. In a nomogram, the predictive efficiency was notably superior to that of traditional clinical features. Related to known models in ovarian cancer with a comparable amount of genes, ours has the highest concordance index; (4) Conclusions: We propose an 11-gene signature prognosis prediction model based on lipid metabolism genes in serous ovarian cancer.

## 1. Introduction

Epithelial ovarian cancer (EOC) is one of the most lethal gynecological malignancies worldwide [1]. It has a high mortality, constituting 3.3% of all malignant diseases and claiming 5.6% of gynecological cancer-related deaths of women in Germany. [2]. Although the prognosis has been improved to a certain degree by surgical treatment, platinum-based chemotherapy, bevacizumab and poly ADP ribose polymerase inhibitors, the 5-year survival of patients with advanced stage EOC is poor at only 20–30% [3,4]. Therefore, when investigating new therapeutic options it is of clinical importance to identify reliable prognostic markers or models to more accurately study their role in the occurrence and development of EOC.

To date, many driver genes have already been identified. For instance, BRCA1, BRCA2, p53, KRAS, PIK3CA, CTNNB1 and PTEN can be implicated in the development of EOC [5]. EPOR is known for its biological effect on tumor growth [6], TTC30A and LRRC8D regulate the expression of host proteins [7], Tfcp2l1 may be involved in the differentiation of ovarian tumor stem cells [8] and the expression of FOXM1 is correlated with chemotherapy resistance and poor prognosis in patients with non-serous epithelial EOC [9].

Recent studies suggest that lipid metabolism disorders may represent important metabolic markers for cancer cells in general. Metabolic reprogramming, including changes in lipid metabolism, can occur in tumor cells and the tumor microenvironment and has impact on the growth, proliferation, invasion and metastasis of cancer cells [10,11,12,13]. Braicu et al. [14] conducted a comprehensive lipidomics analysis on the serum samples of 147 EOC patients and 98 control subjects with benign ovarian tumors or non-tumorous diseases. They revealed that a variety of lipid molecules could act as prognostic markers for EOC, due to their superior efficacy compared to CA125. Furthermore, EOC cells are known to be more aggressive after reprogramming lipid metabolism in an ascites microenvironment. Targeting the signal transduction axis of lipid metabolism can effectively prevent peritoneal metastasis of EOC under experimental circumstances [15]. Niemi et al. [10] showed that changes in lipid metabolism can occur in various stages of EOC and can become intensified in a consistent pattern with advanced cancer stages.

However, the role and the prognostic value of genes related to lipid metabolism in EOC remain to be clarified, since a lack of large-scale EOC sample populations, especially for validation sets, constrains the reliability and validity of previous research results. However, in an era of big data, the emergence of genome-sequencing technologies and data [16,17,18,19] may help in tumor diagnosis and prognosis prediction [20].

Accordingly, we collected genes related to lipid metabolism and constructed molecular subtypes of serous EOC based on lipid metabolism genes according to The Cancer Genome Atlas (TCGA) and the Gene Expression Omnibus (GEO) databases. After this, we established an 11-gene signature prognosis prediction model to validate its performance in a large panel of tumors.

## 2. Results

### 2.1. Molecular Subtypes of Lipid Metabolism-Related Genes

#### 2.1.1. Identification of Two Molecular Subtypes

**Figure 1:**
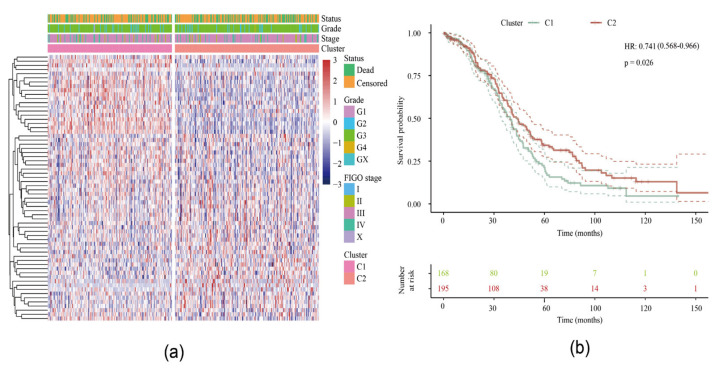
Identification of two molecular subtypes: (**a**) Heat map of clustering of 64 prognosis-related genes. (**b**) Survival curve of molecular subtypes including hazard ratio (HR) with 95% confidence interval (CI) and *p*-value.

After preprocessing, a total of 751 lipid metabolism genes from our serous EOC samples qualified for subsequent analysis (Supplementary Table S1). We then conducted a univariable Cox analysis using the coxph function to obtain 64 prognostic genes (*p* < 0.05). The expression matrix of these genes was obtained and the TCGA samples were divided into two clusters through the non-negative matrix factorization (NMF) algorithm (Supplementary Figure S1). The levels of expression of the 64 genes in the two subtypes are shown in Figure 1a and they differ significantly between cluster 1 (C1) and cluster 2 (C2). Furthermore, the log-rank test showed a significant difference in the overall survival (OS) between these two groups (*p* = 0.026) (Figure 1b) with a better prognosis among C2: 36.82 months in C1 vs. 43.61 months in C2. When we analyzed the disease-specific survival, we were able to confirm a significant difference between both clusters (*p* = 0.033) (data not shown).

#### 2.1.2. Relationship between Two Subtypes and Immunity

**Figure 2:**
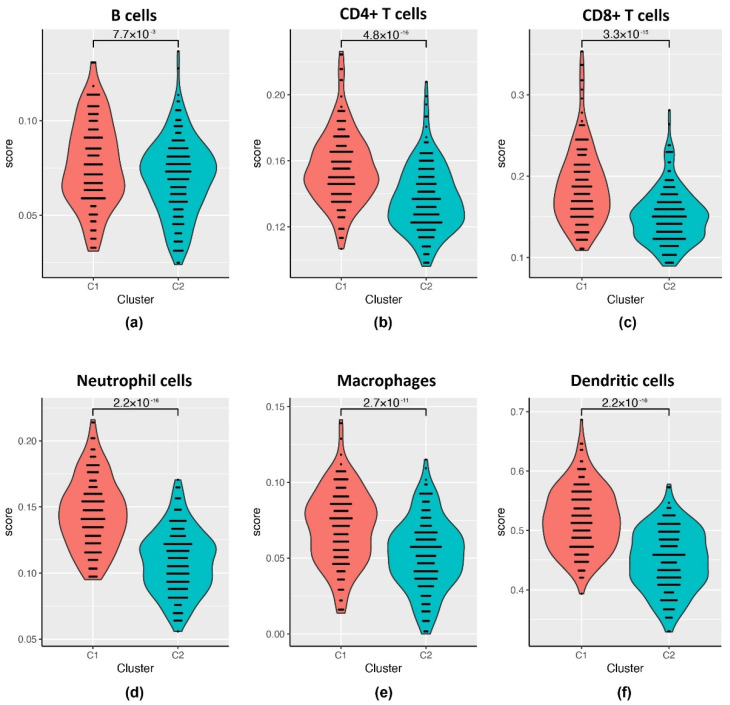
Tumor Immune Estimation Resource (TIMER) immune scores for cluster 1 (C1) and cluster 2 (C2) subtypes with the *p*-value in between for: (**a**) B cells; (**b**) CD4+ T cells; (**c**) CD8+ T cells; (**d**) neutrophil cells; (**e**) macrophages; (**f**) dendritic cells.

It is known that the expression of immune genes is associated with different genomic aberrations in gynecological cancers [21] and Yang et al. [22] showed the dependence of tumor-infiltrating lymphocyte type on different types of EOC. To include an immunological viewpoint on our two clusters we used the Tumor Immune Estimation Resource (TIMER) tool. We then compared the immune scores for six different lymphocytes between C1 and C2 (Figure 2, Supplementary Table S2). Overall, the median immune scores of the six types of lymphocytes are significantly higher in the C1 than in the C2 subtype (*p* < 0.01): B cells 0.076 vs. 0.072, CD4+ T cells 0.152 vs. 0.135, CD8+ T cells 0.181 vs. 0.147, neutrophil cells 0.143 vs. 0.109, macrophages 0.072 vs. 0.053, dendritic cells 0.517 vs. 0.453.

### 2.2. Analysis of DEGs between Subtypes

#### 2.2.1. The DEGs in C2 Subtype Were Mainly Downregulated

A total of 925 differentially expressed genes (DEGs) between the two subtypes were identified (Supplementary Table S3). As shown in Supplementary Figure S2, in C2 subtype, 193 genes were upregulated in comparison to C1, whereas 732 genes were downregulated when compared to C1.

#### 2.2.2. DEGs Are Enriched in Tumor-Related Pathways

**Figure 3:**
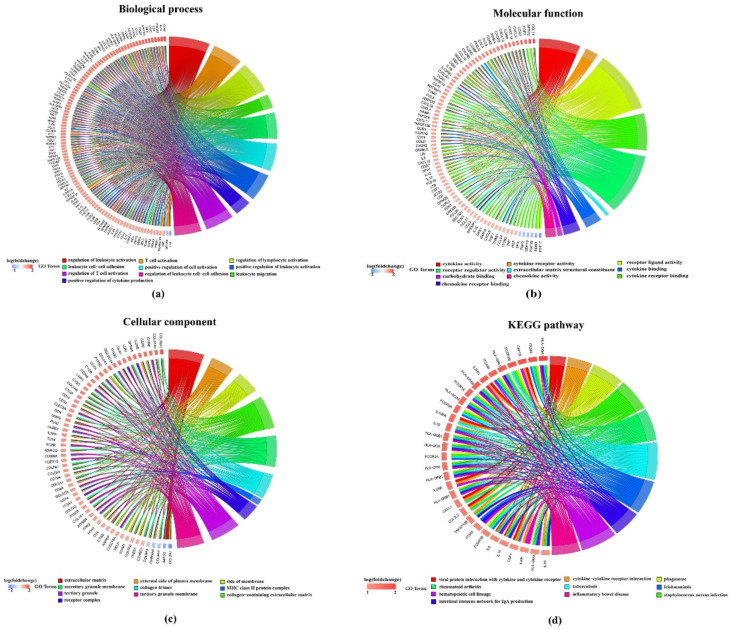
Kyoto Encyclopedia of Genes and Genomes (KEGG) and Gene Ontology (GO) functional enrichment analysis on 925 differentially expressed genes (DEGs) with each top ten annotations in: (**a**) Biological process subsection of GO; (**b**) molecular function subsection of GO; (**c**) cellular component subsection of GO; (**d**) KEGG pathways.

We performed Kyoto Encyclopedia of Genes and Genomes (KEGG) and Gene Ontology (GO) functional enrichment analysis on these 925 DEGs using the clusterProfiler package. The DEGs were collectively enriched to 1871 biological process annotations and there were 749 significant annotations with a false discovery rate (FDR) < 0 (Supplementary Table S4a). For visualization, we selected the top ten functional annotations according to FDR. As shown in Figure 3a, genes are significantly enriched e.g., in regulation of leukocyte activation, T cell activation, regulation of lymphocyte activation and many more. Similarly, 66 significant functional annotations were enriched in the molecular function region (FDR < 0.01) and 58 significant functional annotations were enriched in the cellular component region (FDR < 0.01) (Figure 3b,c, Supplementary Table S4b,c). Under the scope of the KEGG database, DEGs were significantly enriched in 39 pathogenetic pathways, e.g., for rheumatoid arthritis or inflammatory bowel disease (Figure 3d, Supplementary Table S4d).

### 2.3. Construction of a Prognostic Risk Model

#### 2.3.1. Randomly Grouping of Training and Testing Cohort

The final training cohort had a total of 253 samples and the testing cohort had 110 samples in total. The difference between them was analyzed using a chi-square test. The results showed that the grouping was reasonable and no significant differences were found between the groups comparing event rate, Fédération Internationale de Gynécologie et d’Obstétrique (FIGO) stage, age and grade (Table 1). 

#### 2.3.2. Univariable Analysis of Training Cohort

We used the training cohort to conduct a univariable analysis on each gene by using survival coxph function package. A *p*-value less than 0.05 was selected as the threshold. We found 30 prognosis-related significant DEGs (Supplementary Figure S3, Supplementary Table S5).

#### 2.3.3. Construction of the 11-Gene Signature Using Lasso Regression

Lasso Cox regression analysis was performed to compress the 30 genes from Section 2.3.2. As seen in Supplementary Figure S4a, with decreased lambda, the number of independent variable coefficients approaching zero increased gradually. The model is optimal, which means stable, when lambda = 0.0686 (Supplementary Figure S4b). Therefore, we selected 11 genes under the condition of lambda = 0.0686 as the target genes (Supplementary Table S6): PI3, RGS1, ADORA3, CH25H, CCDC80, PTGER3, MATK, KLRB1, CCL19, CXCL9 and CXCL10. These 11 genes were analyzed by multivariate Cox analysis to obtain each coefficient.

#### 2.3.4. Construction and Evaluation of a Risk Model

The 11-gene risk model was established according to the following formula:

$$\begin{array}{l} \mathrm{risk}\;\mathrm{score}\;\left(\mathrm{RS}\right)=\\ \left(0.0004\;*\;\mathrm{expression}\;\mathrm{level}\;\mathrm{of}\;\mathrm{PI}3\right)\\ +\left(0.0044\;*\;\mathrm{expression}\;\mathrm{level}\;\mathrm{of}\;\mathrm{RGS}1\right)\\ +\left(0.0227\;*\;\mathrm{expression}\;\mathrm{level}\;\mathrm{of}\;\mathrm{ADORA}3\right)\\ +\left(0.0103\;*\mathrm{expression}\;\mathrm{level}\;\mathrm{of}\;\mathrm{CH}25H\right)\\ +\left(0.0078\;*\;\mathrm{expression}\;\mathrm{level}\;\mathrm{of}\;\mathrm{CCDC}80\right)\\ +\left(0.0510\;*\;\mathrm{expression}\;\mathrm{level}\;\mathrm{of}\;\mathrm{PTGER}3\right)\\ +\left(0.0357\;*\;\mathrm{expression}\;\mathrm{level}\;\mathrm{of}\;\mathrm{MATK}\right)\\ +\left(-0.0640\;*\;\mathrm{expression}\;\mathrm{level}\;\mathrm{of}\;\mathrm{KLRB}1\right)\\ +\left(-0.0294\;*\;\mathrm{expression}\;\mathrm{level}\;\mathrm{of}\;\mathrm{CCL}19\right)\\ +\left(-0.0020\;*\;\mathrm{expression}\;\mathrm{level}\;\mathrm{of}\;\mathrm{CXCL}9\right)\\ +\left(-0.0006\;*\;\mathrm{expression}\;\mathrm{level}\;\mathrm{of}\;\mathrm{CXCL}10\right). \end{array}$$

**Figure 4:**
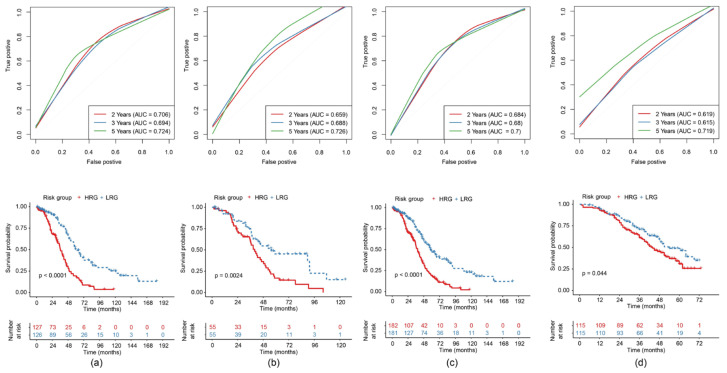
Construction and evaluation of the 11-gene risk model with each receiver operating characteristic (ROC) curve (area under the curve (AUC) of the 2-year, 3-year and 5-year predictive effect) above and the KM (Kaplan-Meier) curve comparing high-risk group (HRG) and low-risk group (LRG) beneath for: (**a**) TCGA training cohort; (**b**) TCGA testing cohort; (**c**) TCGA-(epithelial ovarian cancer (EOC) cohort; (**d**) GEO Series 32026 (GSE32026) cohort.

The risk score (RS) of each sample was calculated and consequently the median RS was applied as the threshold to subdivide the training cohort into a high-risk group (HRG) and a low-risk group (LRG). Considering the overall distribution of the sample’s OS, we evaluated the 2-year, 3-year and 5-year predictive effect of the model. In the receiver operating characteristic (ROC) curve the 5-year area under the curve (AUC) was 0.724 in the training cohort. We observed a significant difference in the Kaplan-Meier (KM) curve between the HRG and the LRG (Figure 4a).

To verify the stability and reliability of the model, we also calculated the prediction performance of this model in the testing cohort for the 2-, 3- and 5-year predictive effect. The result of the testing cohort showed a 5-year AUC of 0.726 and confirmed a significant difference between the HRG and the LRG in the KM curve (Figure 4b). The results of the ROC curve analysis and the KM curve of the whole TCGA-EOC cohort (Figure 4c), as well as the independent external validation through GEO Series 32026 (GSE32026), saw identical results (Figure 4d).

Among these 11 genes, expression levels of PI3, RGS1, ADORA3, CH25H, CCDC80, PTGER3 and MATK were upregulated in the HRG compared with the LRG. In contrast, the expression levels of KLRB1, CCL19, CXCL9 and CXCL10 were upregulated in the LRG, showing a consistent pattern within the training and the validation cohorts (Supplementary Figure S5).

In order to evaluate the stability of the model, we conducted 1000 random samplings at different proportions from all the TCGA-EOC samples. We found significance in 997 out of 1000 times when the sampling ratio was 0.5 (Supplementary Figure S6). This confirmed a lower sampling bias.

### 2.4. Univariable and Multivariable Analysis of Gene Signature

To identify the independence of the 11-gene signature model in clinical application, we conducted univariable and multivariable Cox regression analysis to investigate the relevant hazard ratio (HR), 95% confidence interval (CI) of HR and the *p*-value. We systematically analyzed the clinical information of TCGA patients including age, FIGO stage, grade and our RS of the 11-gene signature (Table 2). Univariable Cox regression analysis found that the RS was significantly related to survival (HR = 1.593, 95% CI: 1.377-1.843, *p* = 3.77E-10). Moreover, the corresponding multivariable Cox regression analysis found that the RS also correlated significantly with survival (HR = 1.534, 95% CI: 1.322-1.780, *p* = 1.65E-08) (Supplementary Figure S7).

### 2.5. Survival Curves of Risk Models in Different Clinical Subgroups

**Figure 5:**
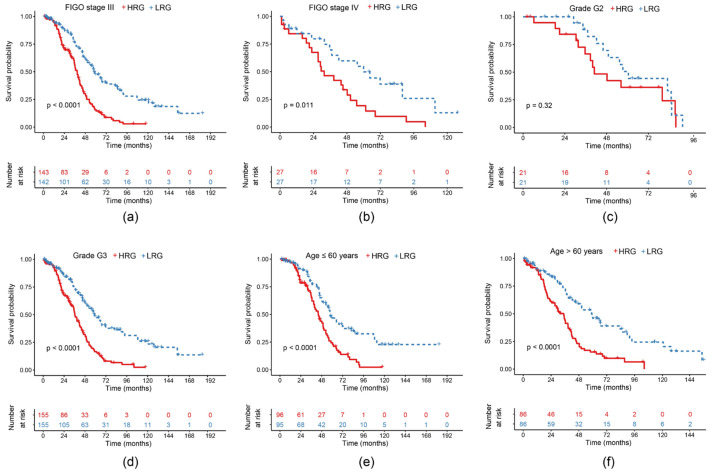
KM curves of overall survival (OS) of the 11-gene risk model in different clinical subgroups: (**a**) Fédération Internationale de Gynécologie et d’Obstétrique (FIGO) stage III; (**b**) FIGO stage IV; (**c**) grade G2; (**d**) grade G3; (**e**) age ≤60 years; (**f**) age >60 years.

In order to verify the effect of our model on clinical subgroup characteristics, we classified the TCGA-EOC cohort according to the different clinical characteristics from Table 1. Significant differences were found between the HRG and the LRG in FIGO stage III and IV (*p* < 0.05) (Figure 5a,b). Due to an insufficient amount of stage I and II samples, we did not analyze them. G1 samples were also not examined because of the lack of data. G2 sample differences between both groups were not significant (Figure 5c), whereas G3 samples showed a significant difference (*p* < 0.01) (Figure 5d). Patient samples ≤60 years as well as >60 years showed a significant difference between the HRG and the LRG (*p* < 0.0001) (Figure 5e,f).

### 2.6. Survival Prognosis on Different Mutation Subtypes in the Risk Model

**Figure 6:**
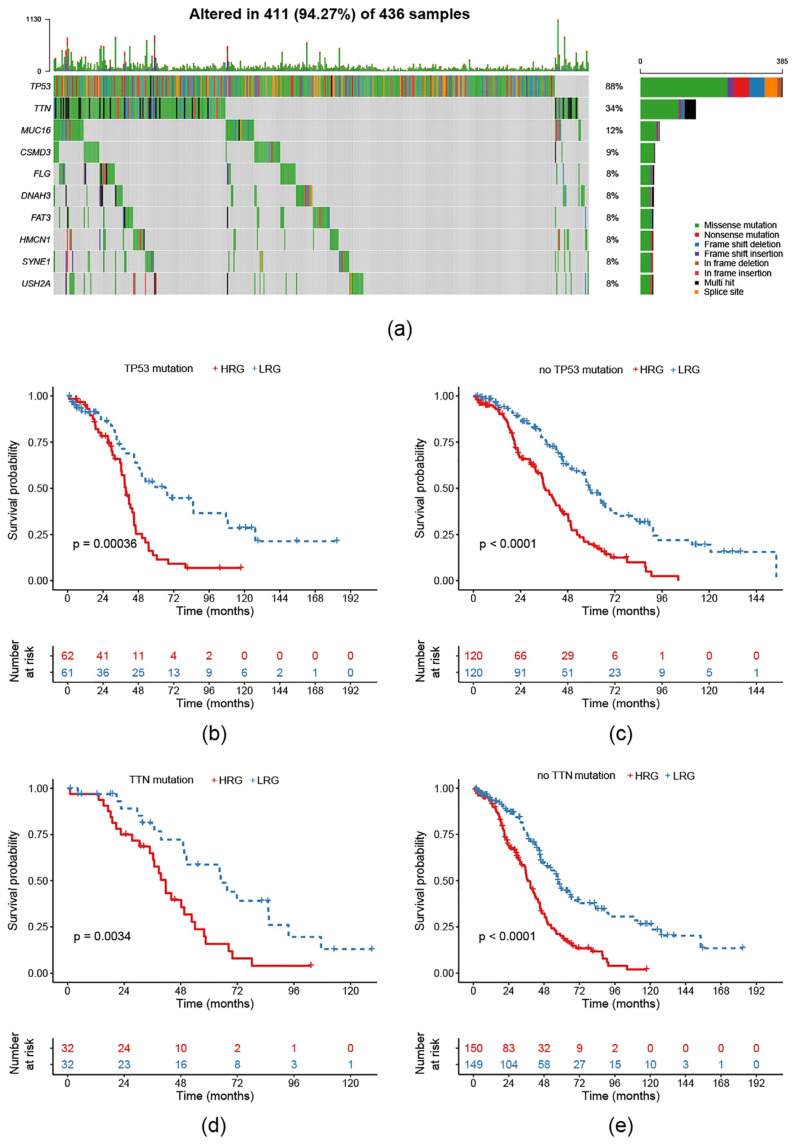
Analysis of the 11-gene signature model in different single nucleotide mutations: (**a**) Distribution of common single nucleotide mutations in EOC with 94.27% samples altered; (**b**) KM curve of TP53-mutated samples; (**c**) KM curve of not TP53-mutated samples; (**d**) KM curve of TTN-mutated samples; (**e**) KM curve of not TP53-mutated samples.

To verify the effectiveness of our model on different common mutation subtypes of EOC, the TCGA-EOC cohort was classified according to different single nucleotide variant types. In TCGA there are a total of 436 exon sequencing samples. Here, we saw 411 altered ones (=94.27%). Mutations of TP53 and TTN dominated this classification (Figure 6a). Consequently, we conducted the KM curves for the 363 RNA-Seq samples and found that, regardless of with/without a TP53/TNN mutation, prognosis in the HRG was worse compared to the LRG (Figure 6b–e).

### 2.7. Construction of Nomogram Model Based on RS and Clinical Features

**Figure 7:**
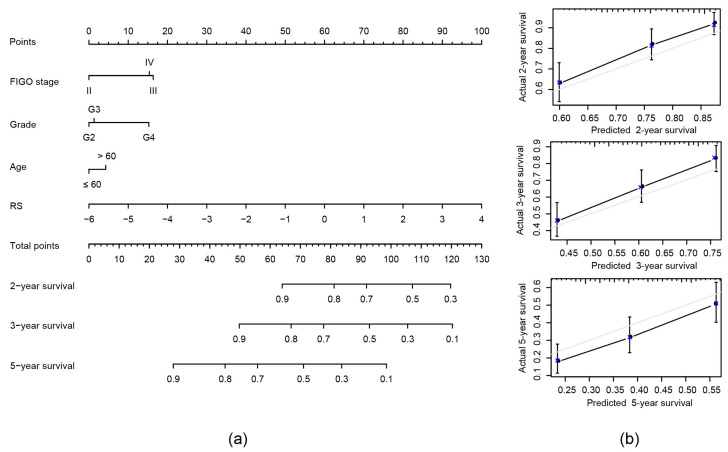
Construction of nomogram model: (**a**) Nomogram predicting 2-, 3- and 5-year OS for patients with EOC. The nomogram is applied by adding up the points identified on the points scale for each variable to a total points amount. Finally, beneath the total points, the probability of 2-, 3- or 5-year survival is projected on the bottom scales. (**b**) Calibration curves for nomogram predicted 2-, 3- and 5-year OS for patients with EOC in relation to actual survival.

We combined the traditional clinical features FIGO stage, age and grade with our RS to construct a nomogram model to predict the OS of EOC patients (Figure 7a). In the modeling results the RS has the greatest impact on survival prediction. Calibration plots were used to visualize the performances of the nomograms. The 2-year, 3-year and 5-year calibration plots demonstrated the performance of our model (Figure 7b).

Under consideration of the nomogram, we saw its notably superior predictive ability compared to clinical features themselves or the RS alone. The concordance index (C-index) of the nomogram was the highest (0.663) compared to the other variables, as seen in Table 3.

### 2.8. ROC Curve and DCA of Nomogram Model

**Figure 8:**
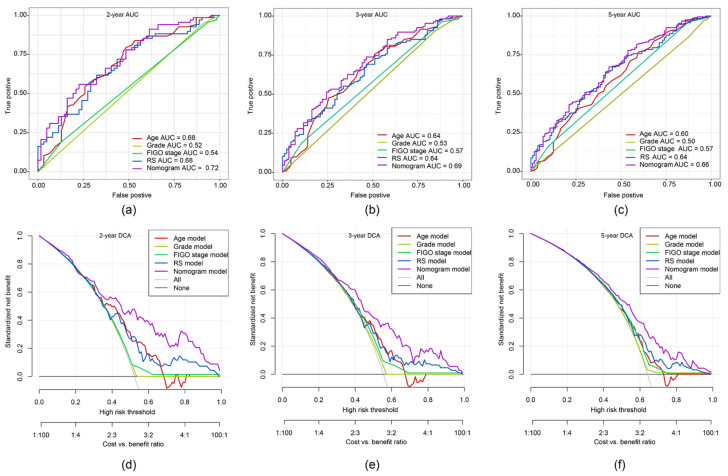
The time-dependent ROC curve and decision curve analysis (DCA) on the nomogram compared to single variable models. Through the ROC curves the accuracy of the models was tested for: (**a**) 2-year survival; (**b**) 3-year survival; (**c**) 5-year survival. The DCA curves can evaluate the clinical benefit of the nomograms and the scope of application. Black indicates that all samples are negative and none are treated, therefore the net benefit is 0. Grey indicates that all samples are positive and all are treated. The x-axis represents threshold probabilities of patients having: (**d**) 2-year survival; (**e**) 3-year survival; (**f**) 5-year survival.

To demonstrate putative advantages of the nomogram model, we compared the 2-, 3- and 5-year ROC curves of the single variables against the nomogram curve. The highest AUC each was seen for the nomogram model (Figure 8a–c). Furthermore, a decision curve analysis (DCA) confirmed our expectations. The net benefit in 2-, 3- and 5-year predictions was the highest in the combined nomogram model compared to the single variable models (Figure 8d–f). These methods showed the improved clinical utility of our nomogram model.

### 2.9. Comparison of the 11-Gene Risk Model with Other Models

**Figure 9:**
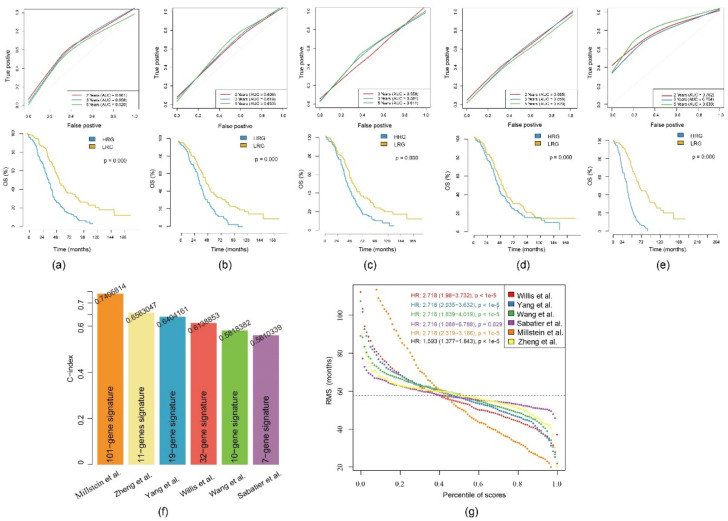
Comparison of the 11-gene risk model with other models: (**a**) The ROC and KM curves of the 19-gene signature (Yang et al.); (**b**) the ROC and KM curves of the 32-gene signature (Willis et al.); (**c**) the ROC and KM curves of the 10-gene signature (Wang et al.); (**d**) the ROC and KM curves of the 7-gene signature (Sabatier et al.); (**e**) the ROC and KM curves of the 101-gene signature (Millstein et al.). (**f**) Concordance indexes (C-indexes) of the six prognostic risk models; (**g**) Restricted mean survival (RMS) time curve of the six prognostic risk models (the dashed line indicates the overlap of 58 months).

Five prognosis-related risk models were selected, including a 19-gene signature from Yang et al. [23], a 32-gene signature from Willis et al. [24], a 10-gene signature from Wang et al. [25], a 7-gene signature from Sabatier et al. [26] and a 101-gene signature from Millstein et al. [27] to compare with our 11-gene model. To ensure comparability, we calculated a RS of the TCGA-EOC cohort for all five models using the same methods as in our gene signature but based on the corresponding genes of each model. As described, samples were divided into a HRG and a LRG with the median as the threshold. The ROC and KM curves of the five models are shown in Figure 9a–e. Only the AUC of Millstein et al. [27] averaged above our model. No significant difference in prognosis was found among the 7-gene signature, whereas all others confirmed significant differences between the HRG and the LRG.

To compare the predictive performance of these models on EOC, we used the restricted mean survival (RMS) package in R [28] to calculate the C-index of all six models including the 11-gene model. The highest C-index was seen in the 101-gene model, while our C-index ranks second (Figure 9f). We used the RMS time to evaluate the predictive effect of the six models at different time points. The RMS curves showed that the six models had an overlap of 58 months. Under the condition of <58 months, our 11-gene risk model performed better than the models from Yang et al. [23], Willis et al. [24], Wang et al. [25] and Millstein et al. [27] (Figure 9g). Thus, our risk model is more suitable to evaluate the data of <5-year OS.

### 2.10. Expression of a Gene Product from the 11-Gene Signature in an EOC Cohort

**Figure 10:**
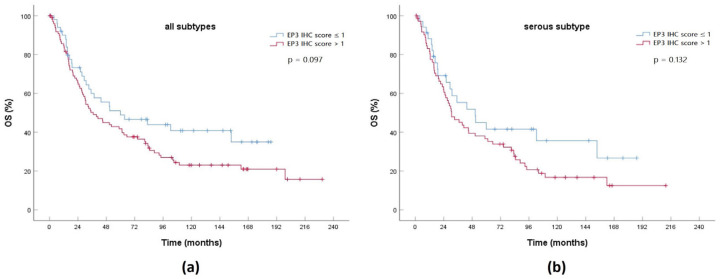
KM curve comparing OS between high prostaglandin E2 receptor 3 (EP3) expression (immunohistochemistry (IHC) score >1) and low EP3 expression (IHC score ≤1) in an EOC cohort for: (**a**) all histological subtypes (*n* = 151); (**b**) serous subtype (*n* = 108).

In a representative EOC cohort from our hospital we measured the expression of the prostaglandin E2 receptor 3 (EP3) encoded by PTGER3, which is upregulated in the HRG compared to the LRG. An immunohistochemistry (IHC) score >1 represents elevated expression of EP3, while an IHC score ≤1 shows low expression. In parallel to the 11-gene signature, higher expression of PTGER3’s gene product EP3 is correlated with poor OS in both the whole cohort and the serous subgroup (Figure 10). Even without having found any significance, this finding supports the functionality of the 11-gene signature and could act as a basis for further confirmation of the 11-gene signature in a clinical context.

### 2.11. Translational Level Validation Related to Signature Genes

**Figure 11:**
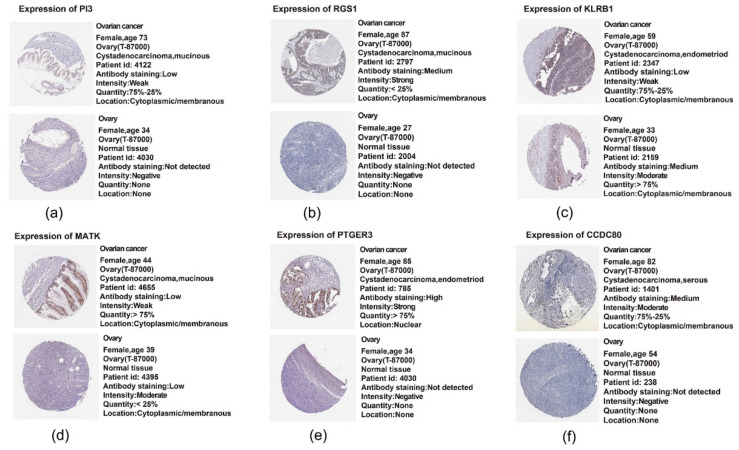
Translational level validation of six genes from the 11-gene signature using the Human Protein Atlas (HPA): (**a**) expression of PI3; (**b**) expression of RGS1; (**c**) expression of KLRB1; (**d**) expression of MATK; (**e**) expression of PTGER3; (**f**) expression of CCDC80.

In order to analyze the translational levels of more signature genes besides PTGER3, the Human Protein Atlas (HPA) database was used. ADORA3, CH25H, CCL19, CXCL9 and CXCL10 were not recorded in the database. The results of PI3, RGS1, CCDC80, PTGER3, MATK and KLRB1 are shown in Figure 11a–f. We found that the expression intensity and quantity of PI3, RGS1, PTGER3 and CCDC80 in ovarian cancer tissue was higher than that in normal ovarian tissue. In contrast, KLRB1’s expression intensity and quantity in normal tissue was higher than that in the tumor tissue. Both findings concur with the expression profile in our 11-gene signature. The expression intensity of MATK in ovarian cancer tissue was lower than that in normal tissue, but had a higher expression quantity, which cannot be clearly correlated with the polarity of our signature.

## 3. Discussion

Due to the lack of early detection and prevention, 70% of EOC patients present in an advanced stage with distant metastases upon diagnosis, making ovarian carcinoma the leading cause of death among malignant gynecological tumors [1,2,29]. Traditional prognostic criteria are not sufficient in accurately predicting the survival of an individual patient. Multiple large cancer databases, such as TCGA and GEO, offer researchers the opportunity to analyze gene expression data and the corresponding clinical information on a large scale [30,31]. Until now, previous studies on gene expression have seen modifications in the molecular signature between benign and malignant tumors or low and advanced tumor stages [32,33,34]. Meanwhile, lipidomic analyses of serum samples have confirmed differences in the lipid profile depending on the tumor’s dignity [14] and have even been shown to prevent peritoneal metastases when targeting the lipid metabolism signaling axis [15].

Consequently, in this study, 363 EOC samples from the TCGA were subdivided, based on 751 lipid metabolism-related genes, into two subtypes. We report that the prognosis of the C1 subtype is significantly poorer than that of the C2 subtype. This finding suggests that lipid-based molecular subtypes can be used, to a certain extent, as an indication for evaluating the prognosis of patients.

To study the individual role of all the genes of both subtypes, we obtained 925 DEGs, of which 193 were upregulated and 732 were downregulated in the C2 compared to the C1 subtype. These genes were mainly active in the regulation of leukocyte activation, T cell activation, regulation of lymphocyte activation and other immunological functions. The immune cell infiltration scores in C1 were found to be significantly higher than in the C2 subtype. Numerous other studies have pointed out the prognostic significance of tumor-infiltrating lymphocytes in other various cancers [35,36,37,38]. It has been reported that tumor infiltration by a subpopulation of CD4+ T cells with immunosuppressive properties predicted reduced survival in EOC [39,40]. Therefore, we can infer that the C1 subtype has a worse prognosis partly because the proportion of CD4+ and CD8+ T cells in the C1 subtype is larger than that in the C2 subtype, as an excessive immune enhancement process might also be a sign of poor prognosis for patients.

Currently, studies on the effect of lipid metabolism on tumor immune functions are being carried out to examine a potential link between both lipids and immune regulation. Interestingly, Wefers et al. [41] discovered that the dysregulation of lipid metabolism in the ascites of EOC patients can affect the immune system by regulating T cell proliferation.

Out of the 925 DEGs, we constructed an 11-gene prognostic risk model based on the genes PI3, RGS1, ADORA3, CH25H, CCDC80, PTGER3, MATK, KLRB1, CCL19, CXCL9 and CXCL10. This model shows a strong robustness and can be used in the prognosis predictions of EOC patients. Calibration plots demonstrated that our nomogram is superior in terms of predictive performance when compared to the grading and FIGO stage. Traditional scores like TNM or FIGO depend on an anatomical spread and, therefore, cannot reflect the biological heterogeneity of EOC [3], which may affect their accuracy.

This is the first prognostic model based on lipid metabolism expression profile. Compared with five other prognostic risk models [23,24,25,26,27] for EOC, the predictive effect of our model at different time points shows that within a survival period of less than 58 months, our 11-gene risk model is the most powerful. Although the model from Millstein et al. [27] has a very high 5-year AUC, it should be noted that their model involves a very large amount of genes, indicating higher costs and consequently reduced clinical utility. Among these 101 genes, the top five genes were TAP1, ZFHX4, CXCL9, FBN1 and PTGER3 (*p* < 0.001), which is interesting because PTGER3 and CXCL9 are in our 11-gene model as well.

While other models did not use the lipid metabolism approach, they also used mainly TCGA data as a base [23,24,25,27]. Only Sabatier et al. [26] used their own patient cohort. In the 19-gene signature, they initially performed a combination with clinical data, which we did as well in the nomogram [23]. In the model from Wang et al. [25] they already evaluated ten biomarkers from the candidate genes and achieved a 100% accuracy. To optimize an early diagnosis of EOC this access via biomarkers remains promising and encouraged us to take a more detailed look into some of the 11 genes selected in our model.

PI3 is located on chromosome 20q 12-13.1 [42,43] and encodes elafin, also known as peptidase inhibitor 3. It is reported to be highly expressed in high-grade serous EOC and is associated with a poor prognosis [44,45]. Wei et al. [46] suggested that elafin selectively regulates the sensitivity of EOC cells to genotoxic drug-induced apoptosis. Our results show that the higher the risk value, the higher the expression of PI3 and the poorer the prognosis of EOC patients, which is consistent with the experimental results.

The regulator of G protein signaling 1 is encoded through RGS1, located on chromosome 1q 31.2 [47]. There is increasing evidence for aberrantly differentiated expression of certain members of this protein family in various cancers and their capability of mediating the proliferation or migration of cancer cells [48]. A study had shown that RGS1 is highly expressed in advanced cervical cancer and is associated with cancer progression [49]. So far, besides our identification of RGS1 as a negative prognosticator in this 11-gene model, no other study reported a role of RGS1 in EOC.

Adenosine receptors are a class of purinergic G protein-coupled receptors with adenosine as an endogenous ligand [50], and ADORA3 codes for one of them. In humans, they are involved in the induction of p53-mediated apoptosis. Consequently, in lung cancer cell lines it is used as a target for antibody-based therapy in p53 mutant tumors [51]. In parallel to RGS1, the biological role of ADORA3 in EOC has not been clarified yet but it should be a target for future research due to its high expression in ovarian tissue, as seen in databases.

The gene product of CH25H is cholesterol 25-hydroxylase, which catalyzes the formation of 25-hydroxycholesterol and thereby results in an inhibitive effect on cholesterol biosynthetic enzymes. It is derived and secreted by U87MG and GM133 glioblastoma cell lines and may be involved in the recruitment of immune-competent cells [52,53]. Mittempergher et al. [54] found that CH25H expression is correlated with the prognosis of breast cancer patients and is an independent predictor of distant metastasis, which is consistent with our data.

The prostaglandin receptor EP3, encoded by PTGER3, is one of the four identified receptors that mediate the effects of prostaglandin E2 [55]. In previous work, our IHC assay showed that EP3 is highly expressed in tissues of clear cell ovarian carcinomas and is a prognostic factor in tumor-associated mucin-1 negative EOC [56]. In this model, high expression of EP3 was grouped into the HRG, indicating poor prognosis, which concurs with the previous experimental results. An analysis of the whole patient cohort from Czogalla et al. [56] for both all histologic subtypes and serous subtype also confirmed the correlation between EP3 as a “high-risk” gene and the clinical data. Additionally to EOC, in our lab, EP3 has been identified as an independent risk factor for the survival prognosis of patients with other gynecological malignancies, such as cervical [57], endometrial [58] and breast cancer [59].

In a meta-analysis of the pan-carcinoma resources and expression characteristics of 18,000 human tumors, Gentles et al. [60] found that KLRB1 is the most favorable pan-cancer prognosis gene and is a marker for enhanced innate immune characteristics in different T cell subsets. Consistent with the results of previous studies, we found that the high expression of KLRB1 was located in the LRG with a good prognosis.

However, several limitations in the current research should be considered. Firstly, the TCGA database is mainly constrained to Caucasian and African populations. Therefore, a robust nomogram should be further validated within multicenter clinical trials and prospective studies. Secondly, we do not have experimental data for the majority of these genes to prove the correlation between the 11-gene signature and the prognosis of EOC. Some of these genes have yet to be reported in the context of EOC. Moreover, we need more external independent datasets to further verify our signature even if we proved the robustness of our signature in the GSE32026 verification cohort. 

A follow-up study to analyze the translational levels of the proteins belonging to these 11 genes is under work. In clinical environments, this gene signature can primarily be used as an additional tool, as it still must be validated in large patient cohorts. In actuality, the signature might support the shared decision-making for or against an incriminating therapy in special patient groups, e.g., very old patients or others with relevant comorbidities. Another aspect in regards to personalized treatment could be the patient’s RS as an indicator for the adaptation of gyneco-oncological aftercare intervals. Finally, biomarkers or therapeutic drugs resulting from our or other gene signatures mentioned would naturally be the biggest therapeutic gain but need a lot of further research. Nevertheless, in an upcoming era of next generation sequencing and gene expression profiling, which we already use as standard critical diagnostic tests in breast cancer treatment, multiple prognostic gene signatures will grow in relevance in our clinical decision-making.

## 4. Materials and Methods

### 4.1. Ovarian Cancer Cohort Source and Preprocessing

The gene expression profiles and clinical follow-up information of EOC were downloaded using the TCGA Genomic Data Commons Application Programming Interface. This cohort contains the expression profile information of 379 RNA-Seq samples of serous EOC. GSE32026 data, downloaded from GEO, covering 260 samples. Human lipid metabolism-related pathways were downloaded from Molecular Signature Database (version 7.0). Herein, a total of 776 lipid metabolism genes (Supplementary Table S7) were sorted out from the six lipid metabolism pathways from the databases KEGG and Reactome (Table 4).

The RNA-Seq data from the TCGA were preprocessed by removing the samples without clinical follow-up information, removing the genes with fragments per kilobase of exon model per million reads mapped less than one and retaining the lipid metabolism gene expression profile. The same procedures were performed on the GSE32026 cohort and the benign tissue samples were removed. A total of 363 samples from the TCGA cohort along with 230 samples from the GSE32026 cohort remained (Table 5).

### 4.2. Identification of Molecular Subtypes Using NMF Algorithm

We extracted the expression of these 776 lipid metabolism genes from the TCGA expression profile data and retained the samples with a gene expression level above zero; 751 genes remained. Univariable Cox analysis was performed using the coxph function in R package to determine the genes that are correlated with the prognosis expressed as OS of EOC (*p* < 0.05). The NMF was used to cluster the EOC samples under the condition of standard NMF “brunet” for 50 iterations by extracting biological correlation coefficients and internal feature structures of the gene expression matrix. The number of clusters k was identified from 2–10. The average contour width of the common member matrix was determined using the NMF package and the minimum member of each subclass was set to 10. We calculated the optimal number of clusters. The selection was based on the following parameters: cophenetic, residual sum of squares and silhouette.

### 4.3. Comparison of Molecular Subtype Immune Scores with TIMER

TIMER is a web server for comprehensive analysis of tumor-infiltrating immune cells [61]. The levels of six tumor-infiltrating immune subsets are precalculated for 10,897 tumors from 32 cancer types. It provides six major analytic modules that allow users to interactively explore the associations between immune infiltrates and a wide spectrum of factors, including gene expression, clinical outcome, somatic mutations and somatic copy number alterations.

### 4.4. Functional Analysis of DEGs

We used the limma package (version 3.42) in R to calculate the DEGs between the different molecular subtypes and filtered these genes in accordance with the threshold of a FDR < 0.05 and |log2(foldchange)| > 1 [62]. Further analysis of the DEGs was performed using clusterProfiler package (version 3.13) [63]. KEGG and GO functional enrichment analysis was conducted. KEGG, a database for the systematic analysis of gene functions, associates genomic information with gene functions and aims to reveal the genetic and chemical blueprint of life. GO enrichment analysis covers three areas including cell components, molecular function, and biological processes.

### 4.5. Sample Preparation

Firstly, the 363 samples in the TCGA cohort were divided into a training cohort and a validation cohort. In order to prevent the bias of random allocation from undermining the stability of the subsequent modeling, all samples were put back into random groups for 100-times in advance. Herein, the group sampling of the training and validation cohorts was performed in the ratio of 7:3. The most suitable training and validation cohorts were selected according to the following conditions:

- The two cohorts are similar in age distribution, FIGO stage, follow-up time and death rate of patients.
- The gene expression profiles of the two data sets that were randomly grouped were close in the number of classified samples.

Finally, the training set had a total of 253 samples and the validation set had 110 samples in total. The difference between the training set and the validation set was analyzed using a chi-square test.

### 4.6. Lasso Regression Analysis

We further compressed the genes using lasso regression to reduce the number of genes in the risk model. The lasso method is a compression estimation used to build a more refined model by constructing a penalty function, thereby compressing some coefficients and setting some coefficients to zero [64]. Therefore, it retains the advantage of contraction in subsets. It is a biased estimation that can be used to process complex collinearity data and can realize the simultaneous selection of variables during parameter estimation to optimize the multicollinearity problem in regression analysis. When applying the glmnet package for lasso Cox regression analysis, we used 3-fold cross validation for model construction and analyzed the CI in each lambda [65]. 

### 4.7. Stability Assessment of Risk Model

In order to evaluate the impact of random sampling on the stability of the model, we conducted 1000 instances of random sampling at various proportions in all TCGA samples to evaluate the times of significant difference in the prognosis of the HRG and the LRG samples.

### 4.8. Construction of Nomogram Combined with RS and Clinical Features

Nomogram is a method to display the results of the risk model intuitively and effectively. It is conveniently applied in the prediction of the outcome. It uses the length of the line to represent the different variables, thereby exhibiting the effect of different variable values on the outcome. We used the TCGA-EOC cohort to build a nomogram that combines FIGO stage, age, grade and RS.

### 4.9. Analysis of DCA

DCA is a simple method to evaluate clinical predictive models, diagnostic tests and molecular markers. It was initially used as a novel analytical technique that incorporated the clinical consequences of a decision to quantify the clinical utility of a prediction nomogram. Therefore, the DCA can decide whether the predictive nomogram is clinically useful or not. The best model is one with a high net benefit as calculated within the favorable probability.

### 4.10. IHC of EP3 in an EOC Patient Cohort

The specimens derived from a cohort consisting of 151 patients with epithelial EOC (serous: *n* = 108, endometrioid: *n* = 20, clear cell: *n* = 11, mucinous *n* = 12) who underwent radical cytoreductive surgery in our department between 1990 and 2002. Diagnoses were done by a specialized gynecologic pathologist. Advanced disease (FIGO IIB-IV) presented in approximately three quarters of the specimens. Mean age at primary diagnosis was 58.9 years. All patients, except FIGO stage IA, received adjuvant platinum-based chemotherapy. Lifetime data were taken from our patient charts, the Munich Cancer Registry and aftercare calendars. The study was carried out in compliance with the guidelines of the Helsinki Declaration of 1964 (last revision October 2018). All participants gave their written informed consent.

The procedure of IHC has been previously described in our lab [57,58,59]. We stained tissue microarrays of the EOC samples of paraffin-embedded and formalin-fixed tissues after epitope retrieval with primary polyclonal anti-EP3 rabbit IgG (Abcam, Cambridge, UK). Afterwards, detection was performed via polymer method with ZytoChem Plus HRP Polymer System mouse/ rabbit (Zytomed Systems, Berlin, Germany) and the chromogen diaminobenzidine (Dako, Hamburg, Germany).

The IHC staining was assessed semiquantitatively, according to Remmele and Stegner [66], using the IHC score. EP3 expression was regarded as negative with an IHC score 0–1 and as positive with an IHC score >1. Expression-dependent differences in OS were tested by chi-square statistic of the Log-Rank test (Mantel-Cox) in KM curves with SPSS Statistics 25 (IBM, Chicago, IL, USA).

### 4.11. Translational Level Validation of Signature Genes

The HPA was initiated in 2003 and shows the expression and localization of human proteins across tissues and organs. It is based on deep RNA-seq from 37 major tissue types and IHC on tissue microarrays containing 44 different tissue types. Altogether, 76 different cell types, covering all major parts of the human body, have been analyzed manually and the data are presented as histology-based annotation of protein expression levels. The antibody-based protein profiles are qualitative and describe the spatial distribution, cell type specificity and the rough relative abundance of proteins in these tissues, whereas the mRNA data provide quantitative data on the average gene expression within an entire tissue. For each gene, the immunohistochemical staining profile is matched with mRNA data and gene/protein characterization data to yield an “annotated protein expression” profile.

## 5. Conclusions

In conclusion, we propose the first 11-gene signature (PI3, RGS1, ADORA3, CH25H, CCDC80, PTGER3, MATK, KLRB1, CCL19, CXCL9 and CXCL10) prediction model based on lipid metabolism-related genes in EOC. Despite different drawbacks of the current analysis, this model may be an interesting approach for a molecular diagnostic test assessing the prognosis and possible risk factors of EOC patients. Furthermore, the development of biomarkers based on this gene signature could represent a perspective for the clinical care of cancer patients.

## Figures and Tables

**Table 1:** Sample information of The Cancer Genome Atlas (TCGA) training and testing cohorts.

Clinical feature		Training Cohort	Testing Cohort	X-Squared	*p*-Value
**Event**	Censored Dead	99 154	42 68	0.0028	0.9575
**FIGO stage**	I II III IV None	0 15 198 37 3	1 5 87 17 0	3.9129	0.4179
**Age**	≤60 >60	132 121	59 51	0.0202	0.8870
**Grade**	G1 G2 G3 G4 None	1 29 217 0 6	0 13 93 1 3	2.7958	0.5926

**Table 2:** Univariable and multivariable analysis of the TCGA cohort.

Clinical Feature	Univariable Analysis			Multivariable Analysis		
	HR	95% CI	*p*-Value	HR	95% CI	*p*-Value
**FIGO stage**	1.929	0.856–4.349	0.1130	1.705	0.742–3.917	0.2080
**Age**	1.186	0.791–1.778	0.4090	1.087	0.718–1.646	0.6940
**Grade**	1.291	0.992–1.680	0.0576	1.193	0.910–1.566	0.2020
**RS**	1.593	1.377–1.843	<0.001	1.534	1.322–1.780	<0.001

**Table 3:** Comparison of concordance indexes (C-indexes) between clinical features, risk score (RS) and nomogram.

Variables	C-Index	95% CI of C-Index	*p*-Value
**FIGO stage**	0.609	0.523–0.696	0.013
**Age**	0.619	0.547–0.690	0.001
**Grade**	0.593	0.508–0.678	0.032
**RS**	0.658	0.602–0.684	<0.001
**Nomogram**	0.663	0.625–0.701	<0.001

**Table 4:** Lipid metabolism-related pathways in the Molecular Signature Database.

Pathway	Database	Gene Count
Peroxisome proliferator activated receptor alpha	Reactome	119
Metabolism of lipids	Reactome	738
Sphingolipid metabolism	Reactome	89
Transcriptional regulation of white adipocyte differentiation	Reactome	84
Glycerophospholipid metabolism	KEGG	77
Fatty acid metabolism	Reactome	77
		Total: 1184 Unique: 776

**Table 5:** Clinical features of the remaining TCGA and GEO Series 32026 (GSE32026) cohort.

Clinical Feature		TCGA	GSE32026
Event	Censored Dead	141 222	116 114
FIGO stage	I II III IV None	1 20 285 54 3	
Age	≤60 >60	191 172	
Grade	G1 G2 G3 G4 None	1 42 310 1 9	

## Supplementary Materials

The following are available online at https://www.mdpi.com/1422-0067/21/23/9169/s1.

## Abbreviations

AUC	area under the curve
C1	cluster 1
C2	cluster 2
C-index	concordance index
CI	confidence interval
DCA	decision curve analysis
DEG	differentially expressed gene
EOC	epithelial ovarian cancer
FDR	false discovery rate
FIGO	Fédération Internationale de Gynécologie et d’Obstétrique
GEO	Gene Expression Omnibus
GO	Gene Ontology
GSE32026	GEO Series 32026
HPA	Human Protein Atlas
HR	hazard ratio
HRG	high-risk group
IHC	immunohistochemistry
KEGG	Kyoto Encyclopedia of Genes and Genomes
KM	Kaplan-Meier
LRG	low-risk group
NMF	non-negative matrix factorization
OS	overall survival
EP3	prostaglandin E2 receptor 3
RMS	restricted mean survival
ROC	receiver operating characteristic
RS	risk score
TCGA	The Cancer Genome Atlas
TIMER	Tumor Immune Estimation Resource
