# Supplementary material for: Development and Validation of a Novel 11-Gene Prognostic Model for Serous Ovarian Carcinomas Based on Lipid Metabolism Expression Profile
Source: Int J Mol Sci. 2020 Dec 1;21(23):9169. doi: 10.3390/ijms21239169 (PMC7731240; doi:10.3390/ijms21239169)
Supplement: Supplementary file 1 [file ijms-21-09169-s001.zip › Supplementary Figure Legends.docx]

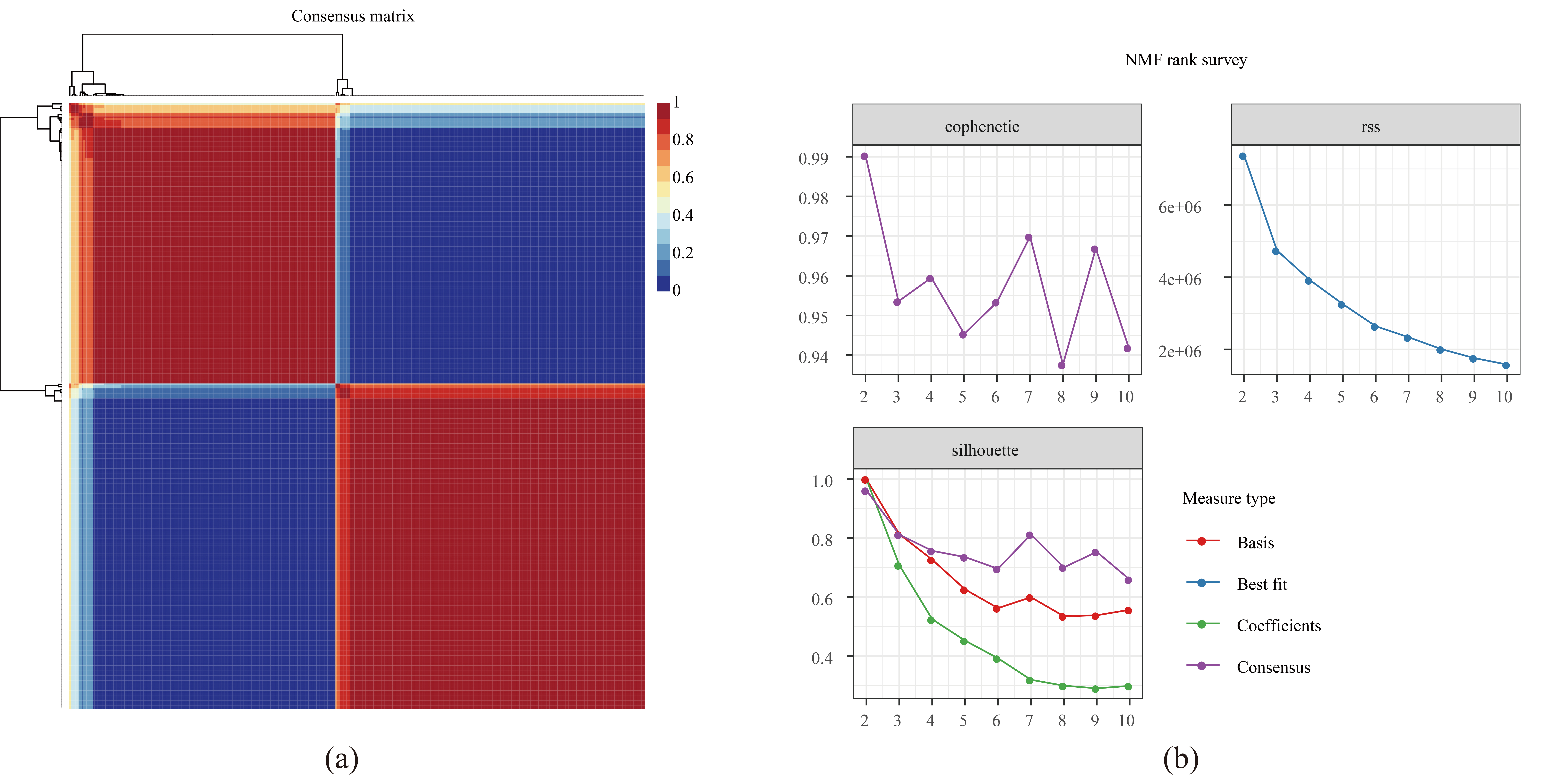


**Supplementary Figure 1.** (**a**) Consensus map of the NMF clustering; (**b**) The distribution of cophenetic, residual sum of squares and dispersion distributions (rank = 2-10). The cophenetic is used to reflect the stability of the cluster obtained from the NMF. The value is between zero and one. The larger the value, the more stable are the clusters. The residual sum of squares is used to reflect the clustering performance of the model. The smaller the value, the better is the clustering effect of the model. Theoretically, the value is the smallest when each sample is clustered into a class. However, such results are not practical and should be combined with other indicators.


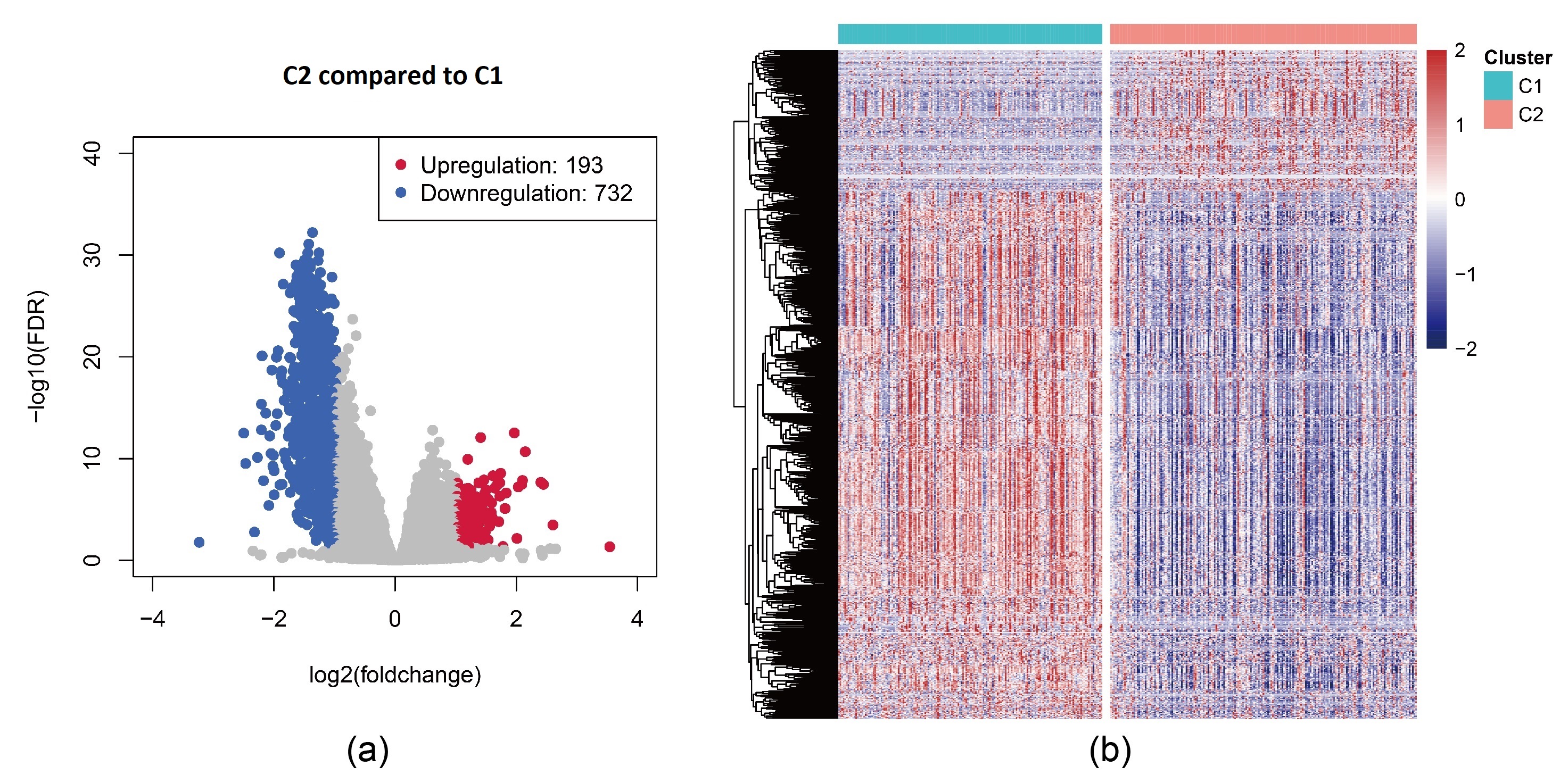


**Supplementary Figure 2.** Identification of DEGs in C2 compared to C1: (**a**) Volcano plot of up- and downregulated DEGs in C2 compared to C1; (**b**) Heat map of DEGs in two subtypes C1 and C2.


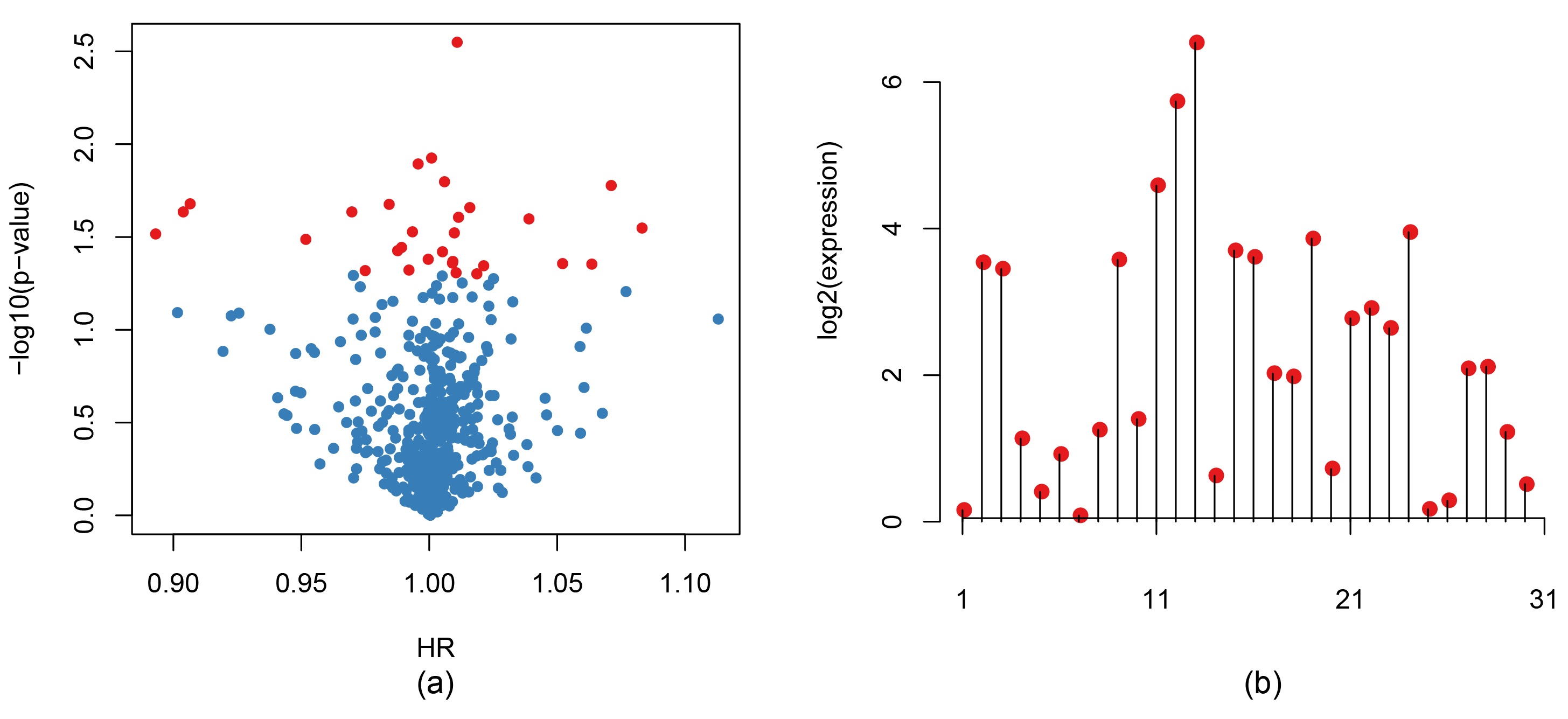


**Supplementary Figure 3.** Univariable enrichment analysis (red dots indicate the genes with log-rank p < 0.05): (**a**) The association between the p-value and HR of 30 significant prognostic DEGs; (**b**) The expression profile of 30 significant prognostic differential genes (log2(expression)).


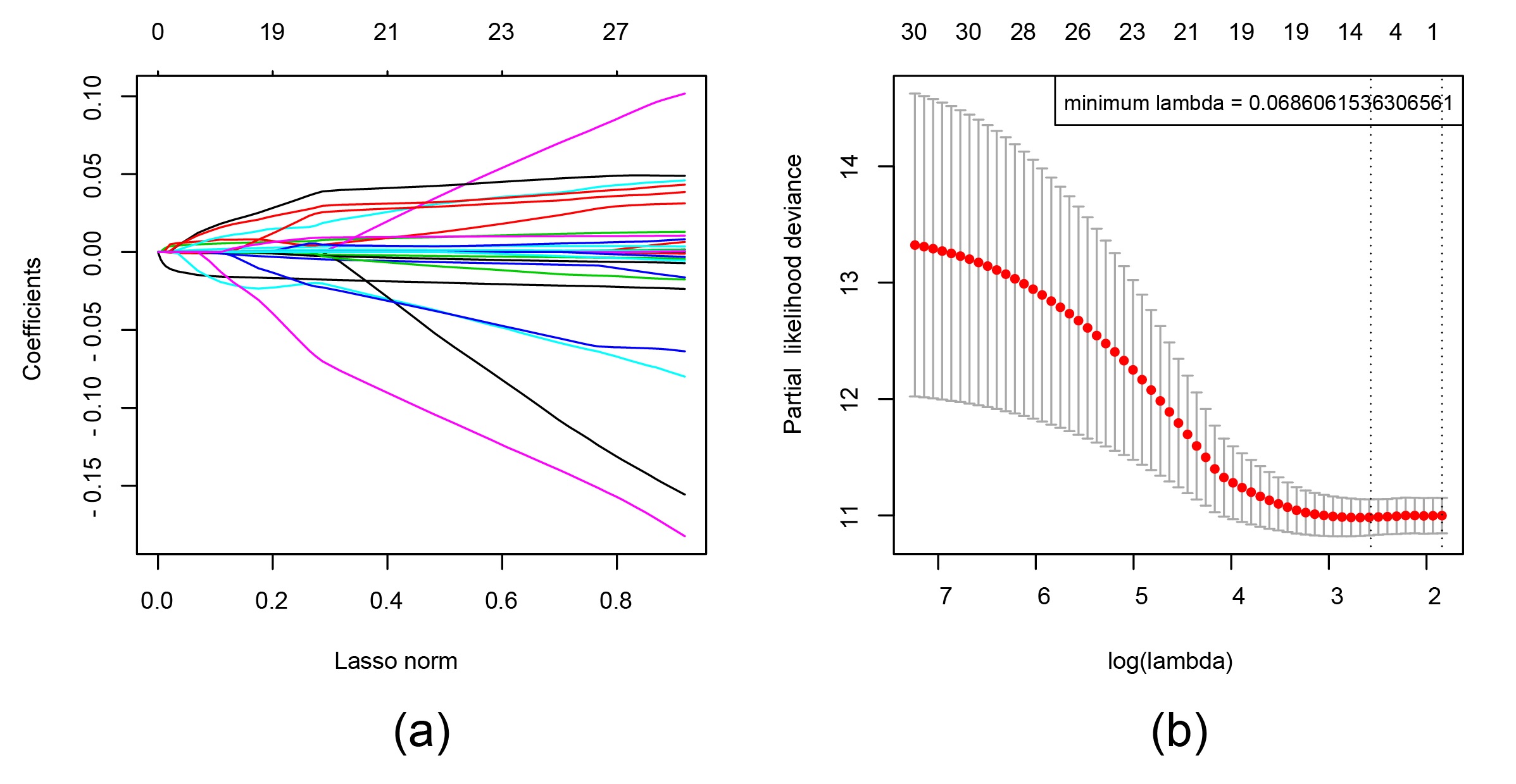


**Supplementary Figure 4.** Analysis of lasso regression: (**a**) The changing trajectory of each independent variable (the abscissa represents the corrected lambda and the ordinate represents the coefficient of the independent variable); (**b**) The log value of the independent variable lambda (the abscissa represents the confidence interval of each lambda and the ordinate represents errors in cross validation).


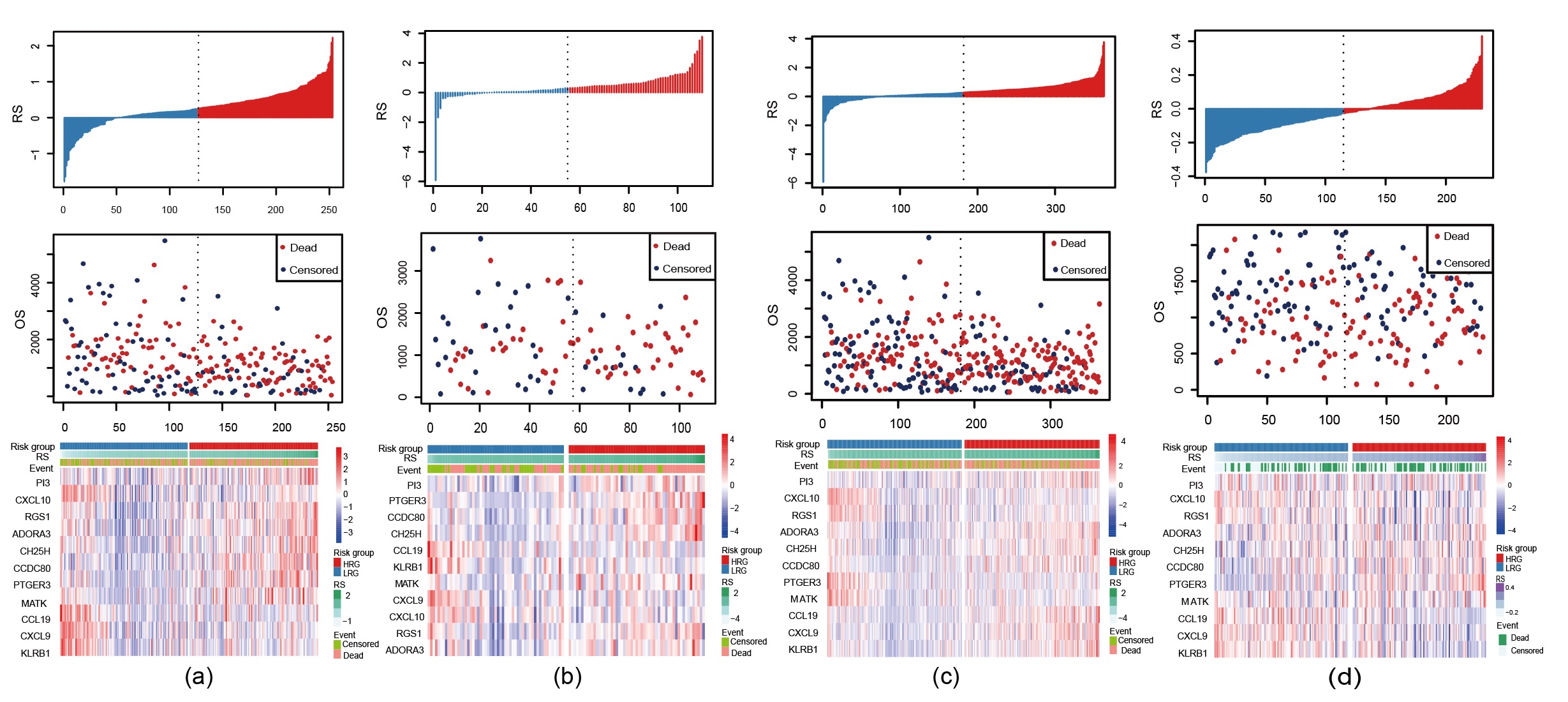


**Supplementary Figure 5.** RS, survival status and the 11-gene expression profile in the: (**a**) TCGA training cohort; (**b**) TCGA testing cohort; (**c**) TCGA-EOC cohort; (**d**) GSE32026 cohort.


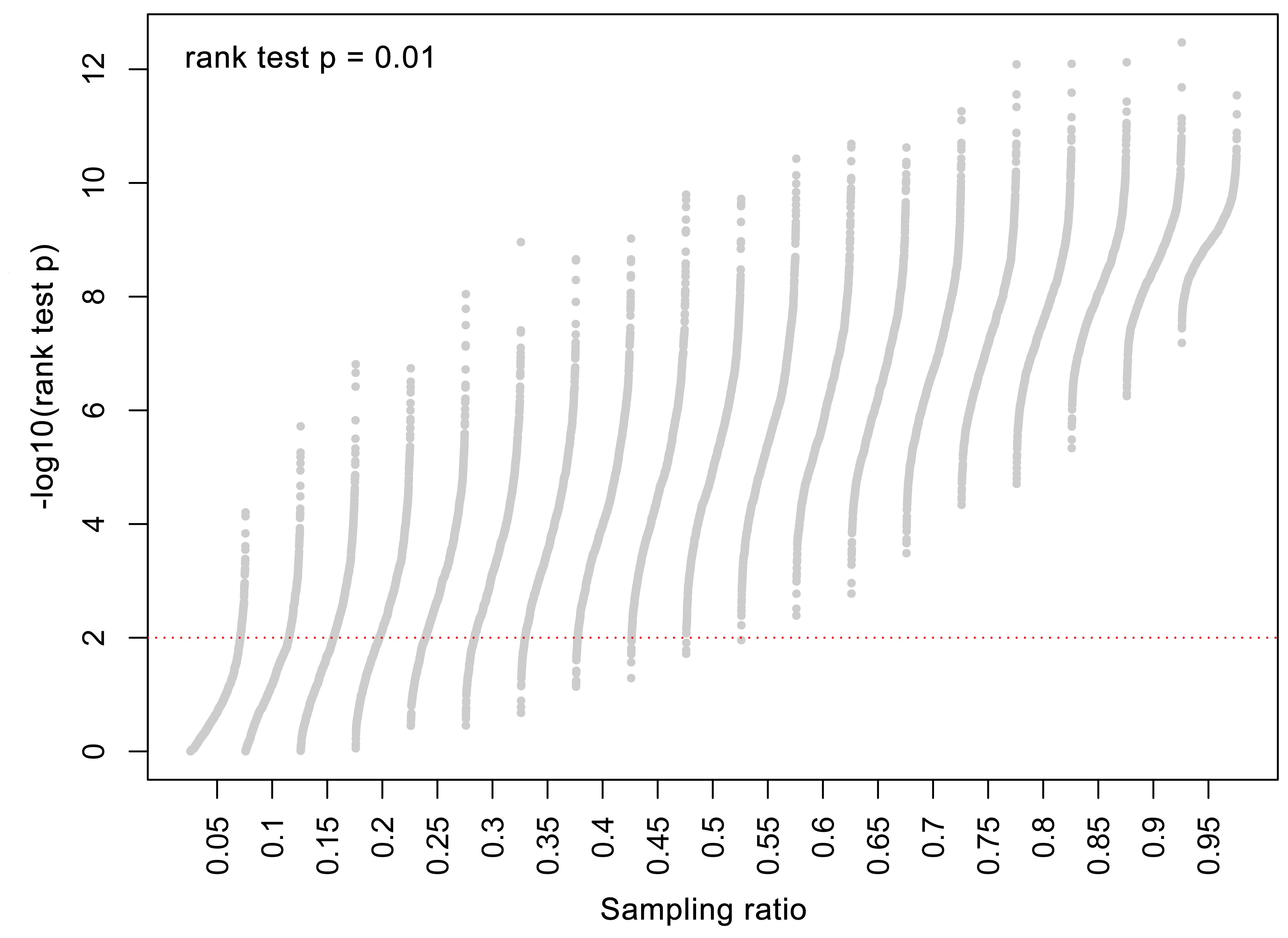


**Supplementary Figure 6.** Stability evaluation of risk model: the distribution of p-values in log-rank test of 1000-times of random sampling at different proportions (the prognostic significance test threshold was set to p < 0.01).


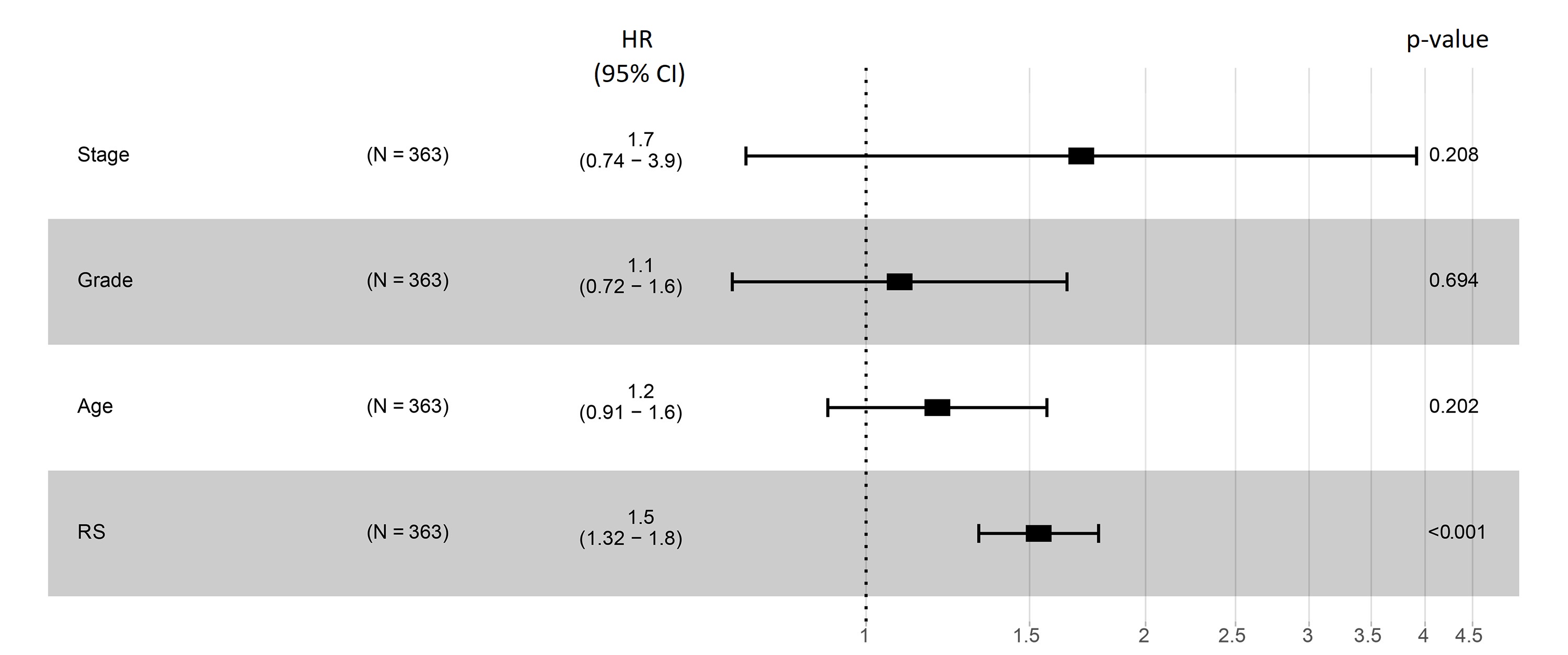


**Supplementary Figure 7.** Forest plot of multivariable analysis: COX regression analysis found that the RS significantly correlated with survival (HR = 1.534, 95% CI: 1.3224-1.7800, p = 1.65E-08).
