# Supplementary figures and images for: Development and Validation of a Novel 11-Gene Prognostic Model for Serous Ovarian Carcinomas Based on Lipid Metabolism Expression Profile
Source: Int J Mol Sci. 2020 Dec 1;21(23):9169. doi: 10.3390/ijms21239169 (PMC7731240; doi:10.3390/ijms21239169)

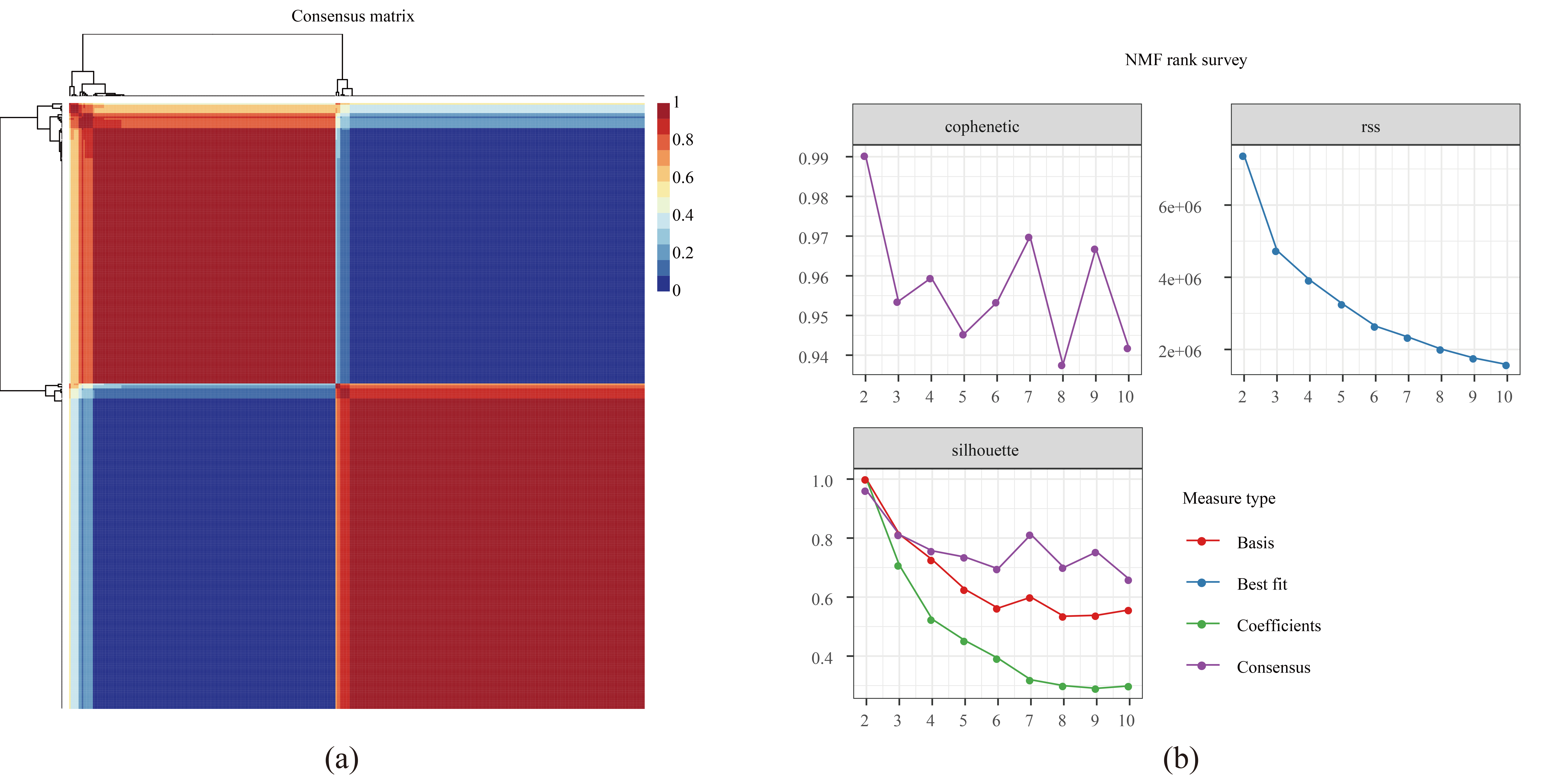

Supplement: Supplementary file 1 [file ijms-21-09169-s001.zip › Supplementary Figure 1.jpg]

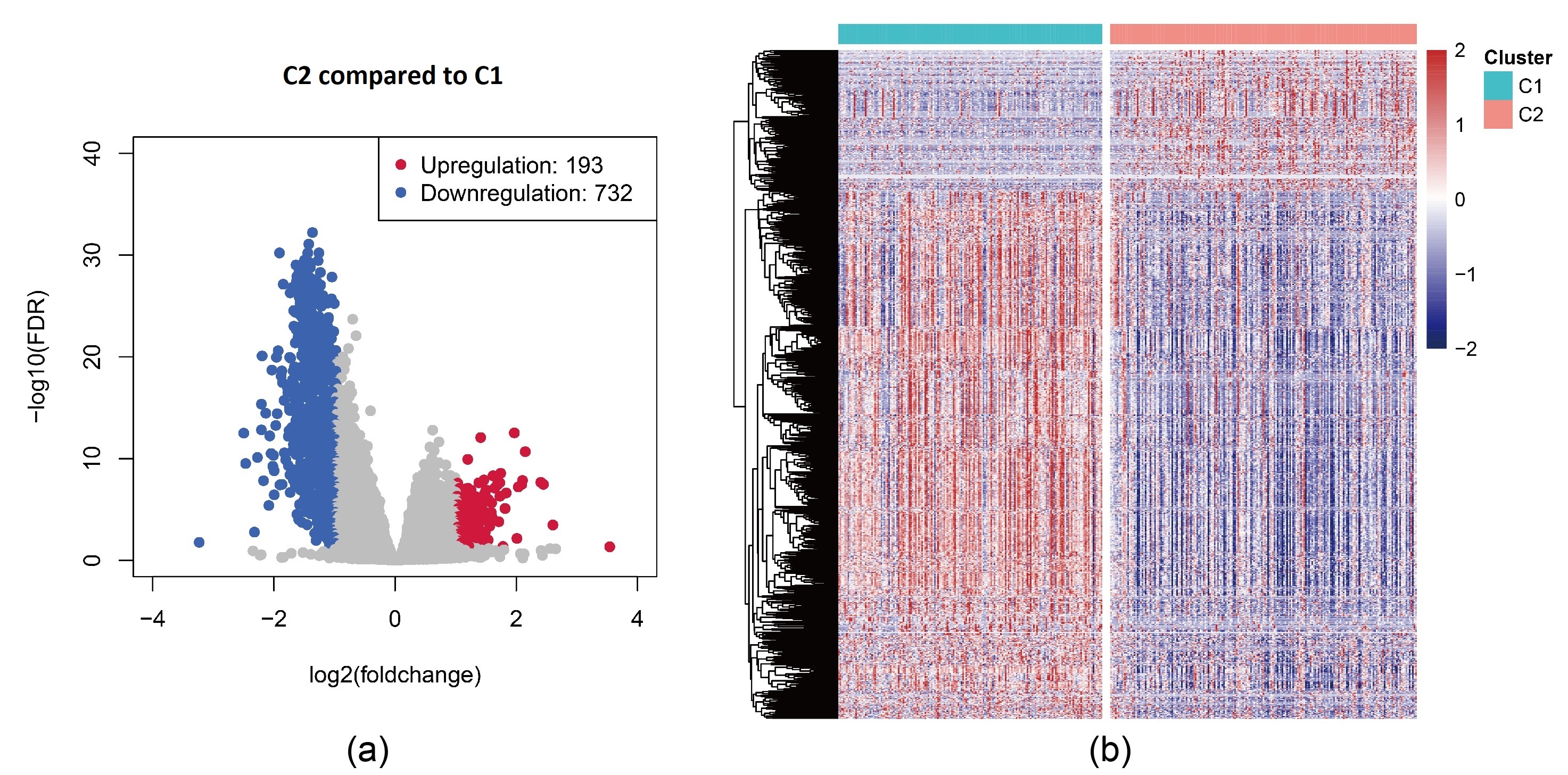

Supplement: Supplementary file 1 [file ijms-21-09169-s001.zip › Supplementary Figure 2.jpg]

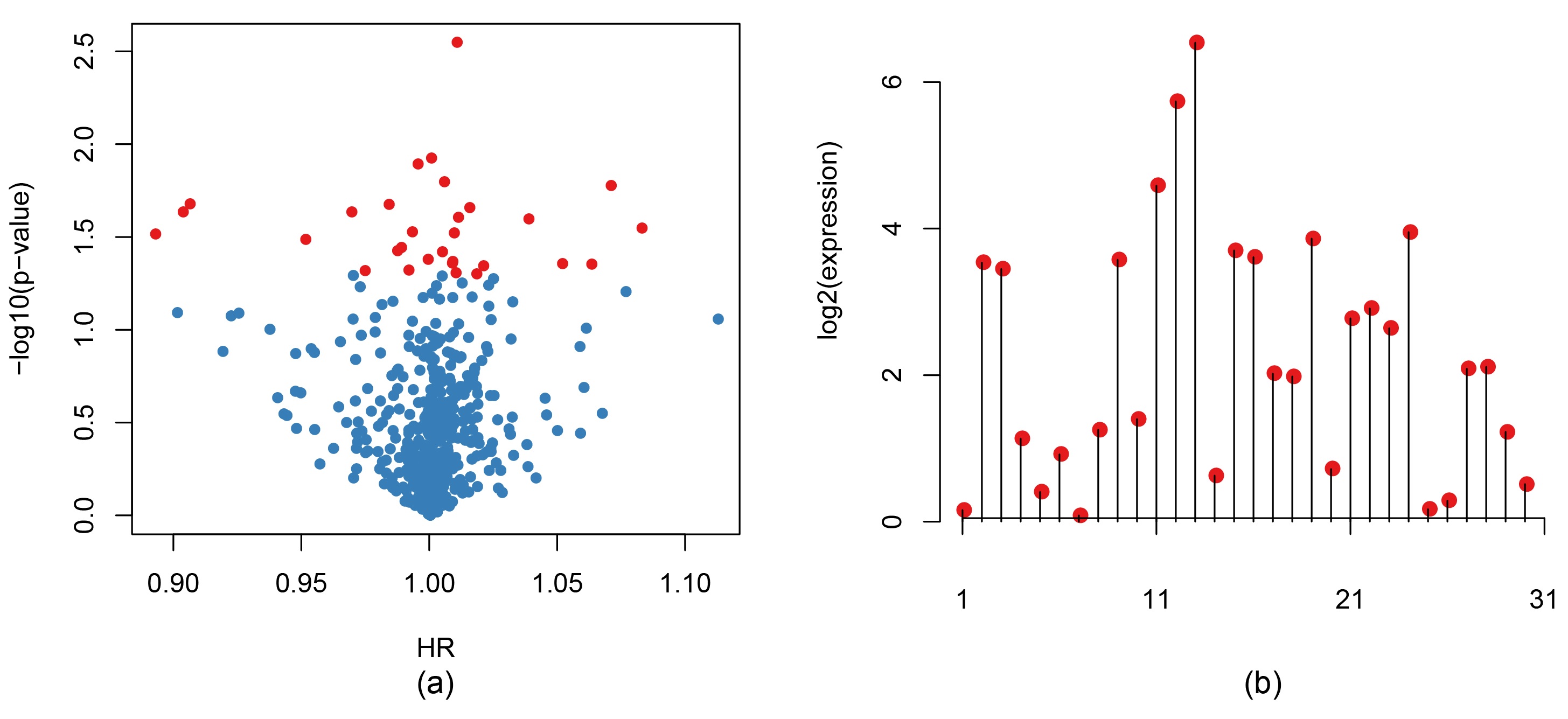

Supplement: Supplementary file 1 [file ijms-21-09169-s001.zip › Supplementary Figure 3.jpg]

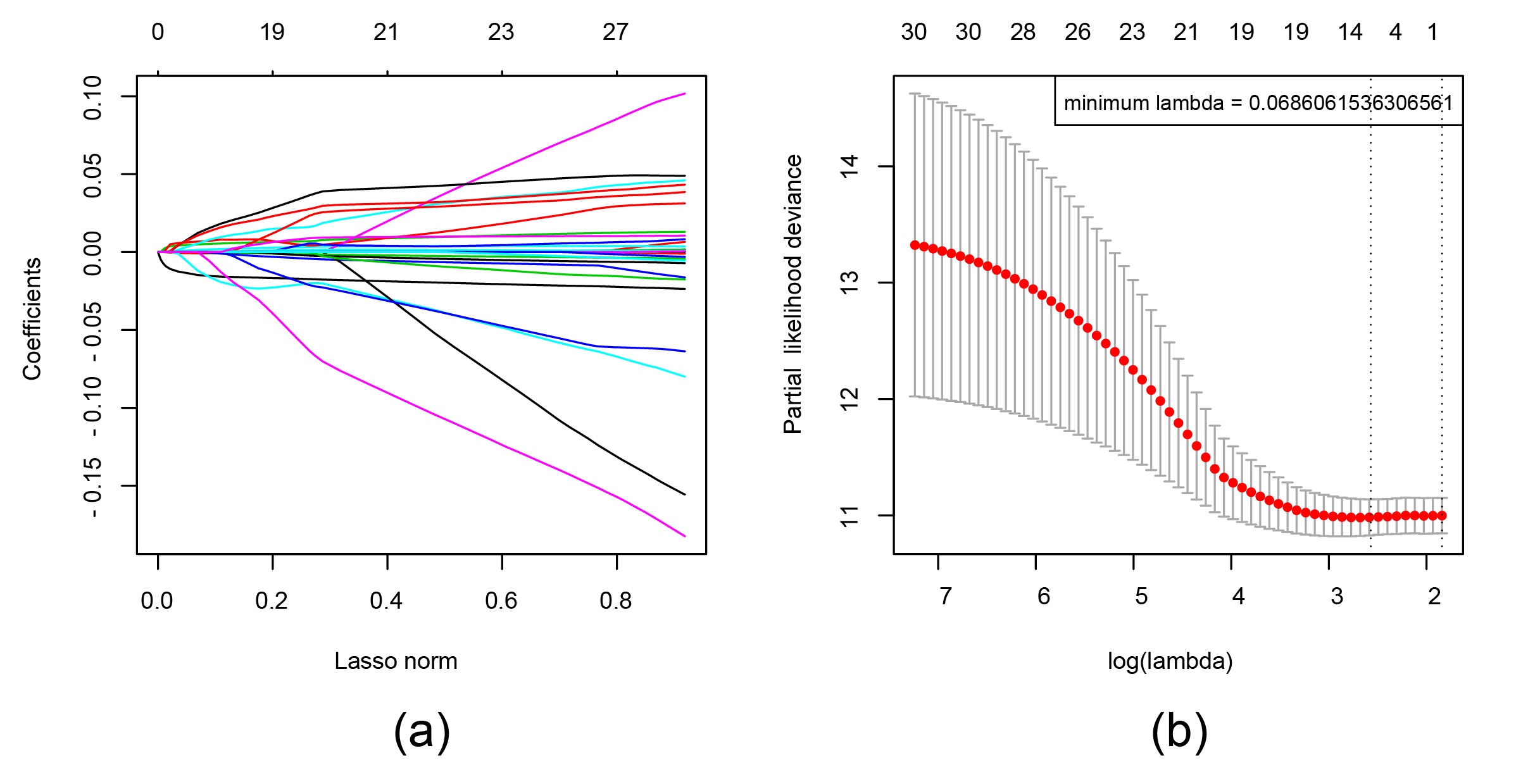

Supplement: Supplementary file 1 [file ijms-21-09169-s001.zip › Supplementary Figure 4.jpg]

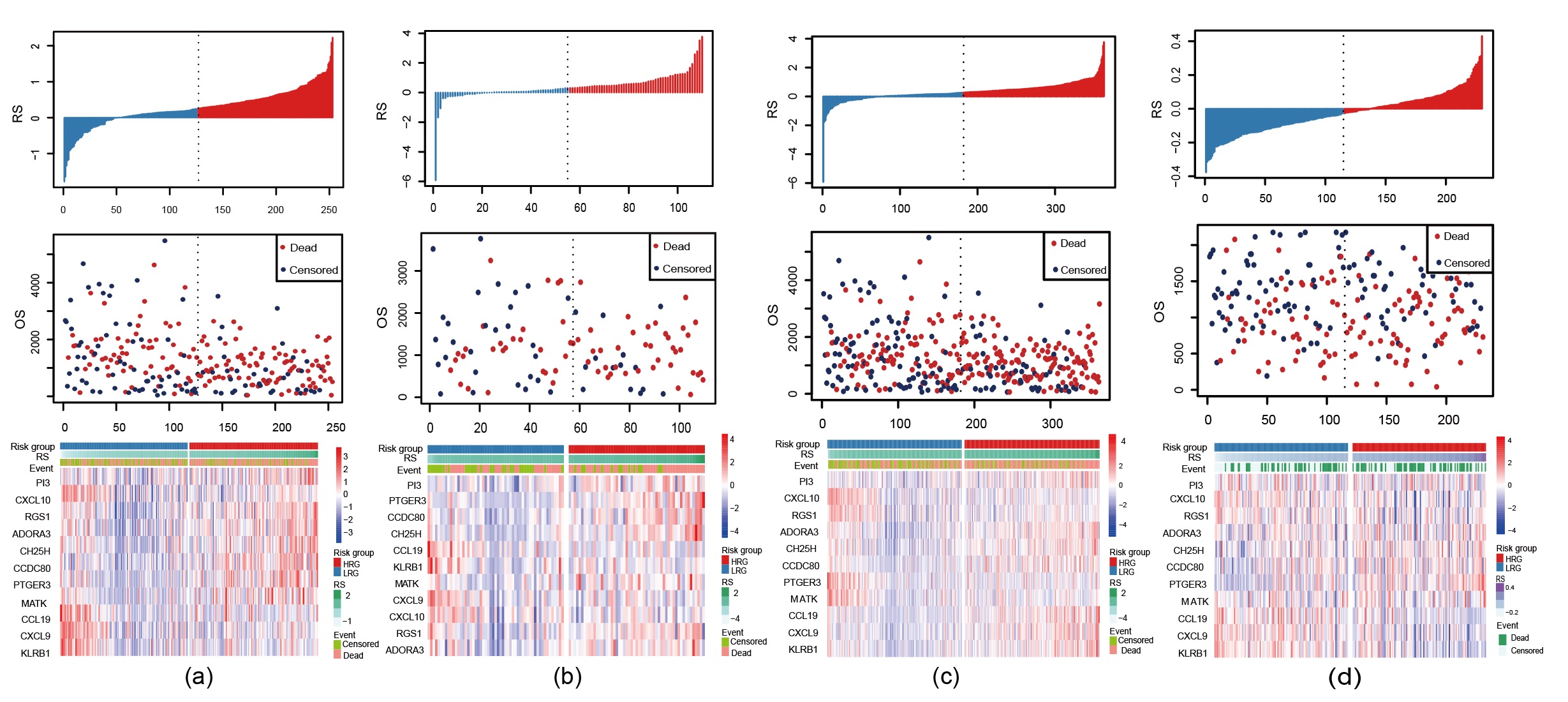

Supplement: Supplementary file 1 [file ijms-21-09169-s001.zip › Supplementary Figure 5.jpg]

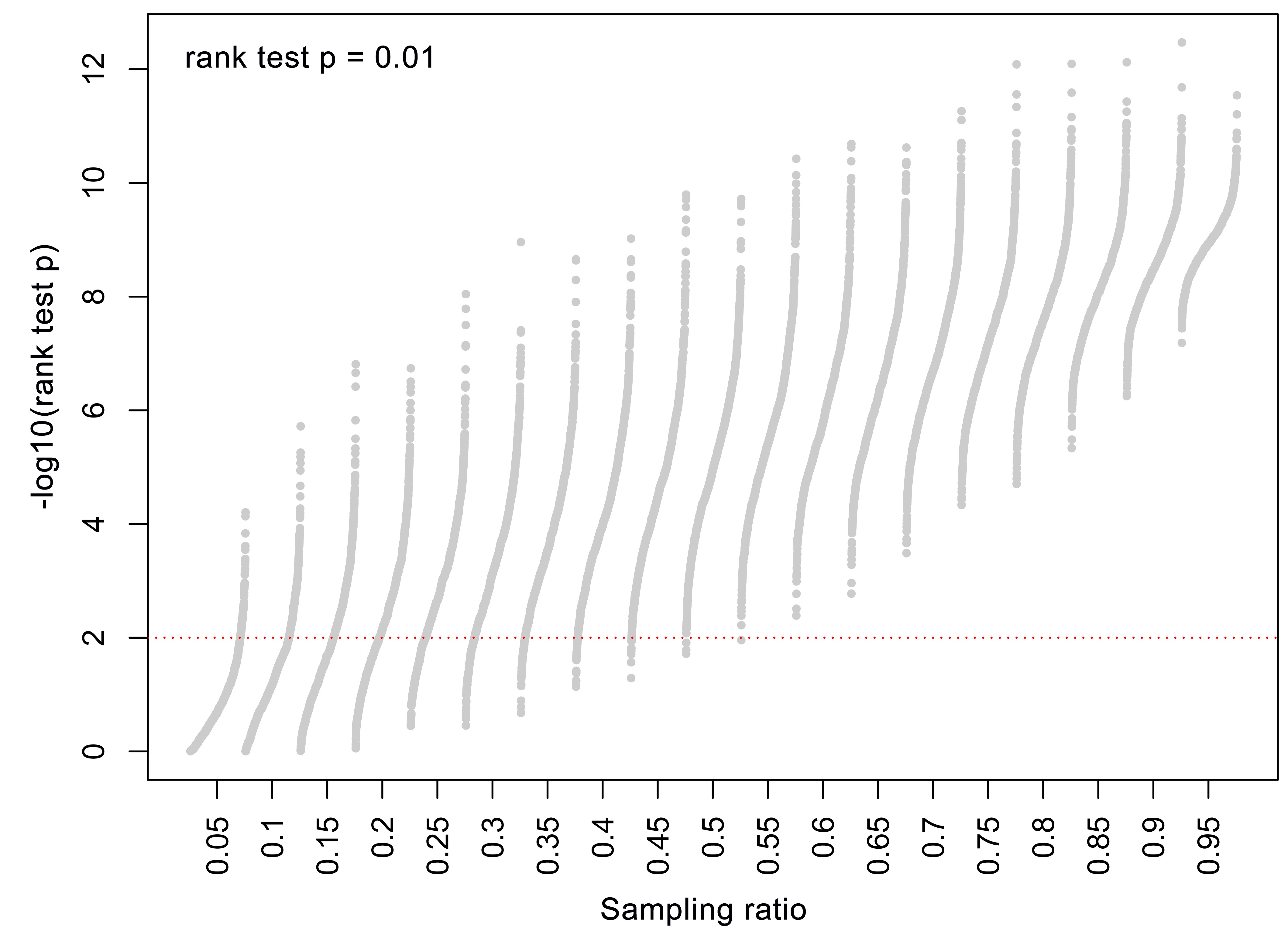

Supplement: Supplementary file 1 [file ijms-21-09169-s001.zip › Supplementary Figure 6.jpg]

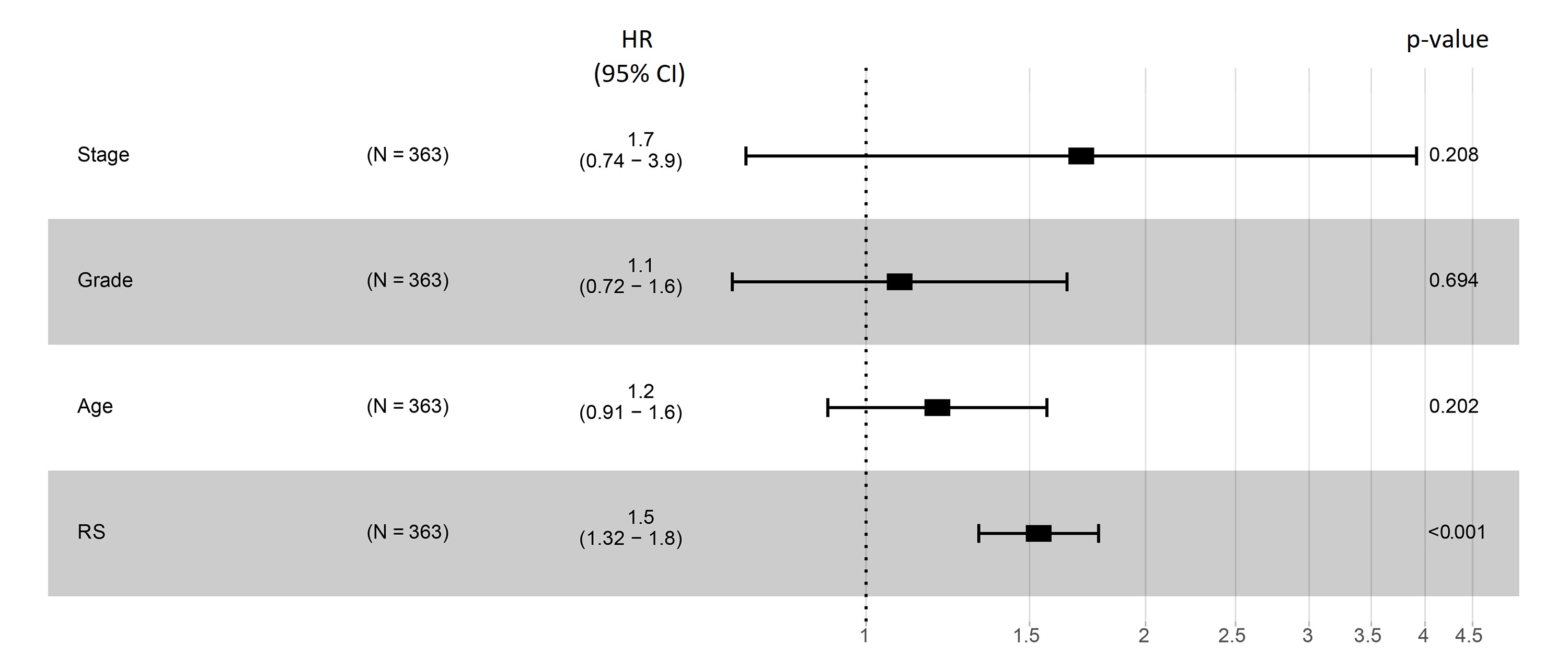

Supplement: Supplementary file 1 [file ijms-21-09169-s001.zip › Supplementary Figure 7.jpg]
